# Role of oral and gut microbiomes in enterosalivary nitrate metabolism and their effects on systemic disease

**DOI:** 10.3389/fcimb.2025.1612223

**Published:** 2025-07-03

**Authors:** Zi Yang, Conglin Du, Zhichao Chang, Yang Yang, Liang Hu

**Affiliations:** ^1^ Department of Endodontics, School of Stomatology, Capital Medical University, Beijing, China; ^2^ Department of Oral and Maxillofacial & Head and Neck Oncology, School of Stomatology, Capital Medical University, Beijing, China; ^3^ Outpatient Department of Oral and Maxillofacial Surgery, School of Stomatology, Capital Medical University, Beijing, China

**Keywords:** nitric oxide, nitrate, nitrate reductase, oral microbiome, gut microbiome

## Abstract

Nitrate, which maintains hemostasis in systemic circulation, is obtained from nitrate-rich vegetables, concentrated, reabsorbed by the salivary glands, and reduced to nitrite and nitric oxide (NO•). The bioavailability of nitrate and nitrite depends on unique nitrate reductases present in specific bacterial communities in the mouth and gut of mammals. The dominant bacteria in the oral cavity, stomach, and gut differ among internal environments. Nitrate can modulate microbiota metabolism and has important pathophysiological functions in diseases such as cardiovascular diseases, gastrointestinal diseases, diabetes, metabolic diseases, and brain diseases via nitrate-reducing bacteria. Thus, in this review, we summarized the beneficial role of enterosalivary nitrate metabolism, focusing on the role of oral and gut bacterial communities in the enzymatic reduction of nitrate to nitrite. We have also discussed different nitrate-reduction pathways; influencing factors of nitrate-reducing bacteria; and the relationship among systemic health, nitrate intake, and bacteria. This review of enterosalivary nitrate and related microbiomes could provide a new perspective for the application of nitrate.

## 1 Introduction

Nitric oxide (NO•) is a gaseous and lipophilic free radical that acts as a signaling molecule and has numerous physiological functions in mammals (Doel et al., 2005). The production and/or bioavailability of NO• is associated with systemic diseases (Lundberg et al., 2015; Stojanovic et al., 2015; Briskey et al., 2016). NO• can be produced from L-arginine by three different nitric oxide synthases (NOSs): neuronal NOS (nNOS), endothelial NOS (eNOS), and inducible NOS (iNOS), via NADPH and oxygen consumption (Knowles and Moncada, 1994; Alderton et al., 2001; Forstermann and Sessa, 2012). Nitrate and nitrite anions are physiologically recycled into NO• and other bioactive nitrogen oxides *in vivo*, serving as an important additional source of NO• independent of NO synthases, especially under hypoxic conditions (Lundberg et al., 2008; Oliveira-Paula et al., 2019). Nitrate supplementation activates the NO_3_^–^ NO_2_^-^ -NO• pathway, which promotes endothelial function, modulates inflammation, protects against ischemia reperfusion injury, supports gastric and mucus formation, enhances exercise capacity, and regulates blood pressure (Petersson et al., 2007; Bailey et al., 2009; Vanhatalo et al., 2010; Bahra et al., 2012; Hobbs et al., 2013; Coggan et al., 2015; Wightman et al., 2015; Briskey et al., 2016; D’El-Rei et al., 2016; Gee and Ahluwalia, 2016; Velmurugan et al., 2016; Munzel and Daiber, 2018; Raubenheimer et al., 2019; Srihirun et al., 2020; Jones et al., 2021).

In mammals, nitrate is directly reduced to nitrite by native xanthine oxidase (XO) in muscles (Piknova et al., 2015, Piknova et al., 2016). However, germ-free animals have negligible levels of gastric NO• even after dietary nitrate loading (Petersson et al., 2015). The use of chlorhexidine (CHX) mouthwash eliminates commensal oral bacteria, resulting in decreased nitrite levels in the saliva, plasma, and urine and increased blood pressure in healthy individuals, suggesting an important role of nitrate-reducing bacteria in the oral cavity of humans (Petersson et al., 2009; Kapil et al., 2013; Hyde et al., 2014b). Thus, a major reduction in nitrate requires enzymes possessed by specific bacteria in the mammalian mouth and gut and some contribution from tissue XO enzyme systems (Doel et al., 2005; Hyde et al., 2014a, Hyde et al., 2014b; Koch et al., 2017; DeMartino et al., 2019).

The oral cavity and gut harbor over 1000 different bacterial species (Nicholson et al., 2005). In the gut, bacterial nitrate reduction and related NO• formation may be an essential aspect of enterosalivary nitrate metabolism (Tiso and Schechter, 2015; Rocha et al., 2016). Despite their important role, nitrate-reducing oral and gastrointestinal bacteria remain uncharacterized, and little is known about the nitrate reduction pathways that are expressed in bacterial species in diverse local environments. Systemic health is associated with the enzymatic reduction of dietary nitrate by nitrate-reducing bacteria. Similarly, there is limited information about the roles of oral and enteric nitrate-reducing bacteria in the control of systemic diseases and the influencing factors in different individuals.

In this review, we discussed and summarized studies that highlight the beneficial role of dietary nitrate intake and the conversion of nitrate and nitrite, which are essential for systemic health, with a particular focus on the role of oral and intestinal microbiota in the reduction of nitrate to nitrite. Different nitrate-reduction pathways in different bacterial species and factors influencing nitrate-reducing microbiomes have also been discussed. In addition, we summarized the relationship between systemic health, nitrate intake, and nitrate-reducing bacteria.

## 2 Enterosalivary nitrate circulation

**Figure 1:**
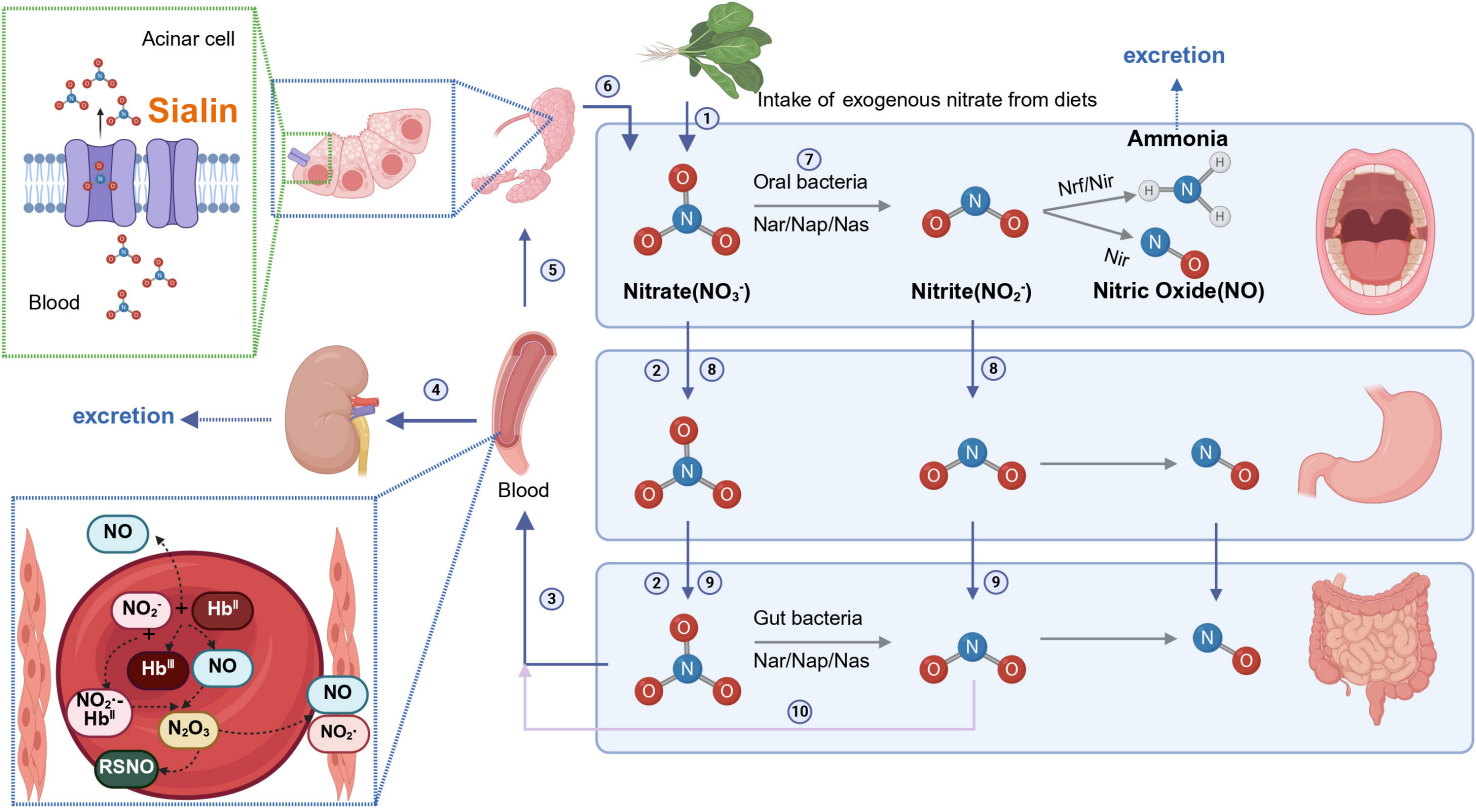
Entero-salivary nitrate circulation (Created with BioRender.com). ① Exogenous nitrate is obtained via dietary intake. ② Dietary nitrate enters the stomach and gut. ③ Dietary nitrate is absorbed through the small intestinal tract to blood. ④ The plasma nitrate (70%–75%) is excreted in the urine. ⑤ Circulating nitrate is actively taken up by the salivary glands via sialin. ⑥ Nitrates flow into the mouth through saliva. ⑦ Salivary nitrate is reduced to nitrite by special commensal bacteria in the mouth. ⑧ Salivary nitrate and nitrite enter the stomach. ⑨ Salivary nitrate and nitrite enter the gut and nitrate is reduced to nitrite by gut bacteria. ⑩ Nitrite is absorbed into blood and nitrite is reduced to NO in the blood. Methemoglobin (metHb) reacts with nitrite to form a radical NO_2_-bound ferrohaem, which reacts rapidly with NO• to generate N_2_O_3_, responsible for S-nitrosothiol (RSNO) formation.

Enterosalivary nitrate circulation is shown in **Figure 1**. Systemic circulating nitrate is mainly obtained from the diet (Archer, 2002; Weitzberg and Lundberg, 2013; Babateen et al., 2018; Ma et al., 2018). Green leafy vegetables, such as spinach and beetroot, are the main nitrate sources (approximately 80%) in the majority of human diets (Babateen et al., 2018). Other sources of nitrate intake include drinking water (15%) and other foods (5%) (Sindelar and Milkowski, 2012).Dietary nitrate enters the stomach and is absorbed through the small intestinal tract into the bloodstream. Approximately 70%–75% of plasma nitrate is excreted in urine. The remaining 20-25% of circulating nitrate is actively concentrated by the salivary glands via sialin, an electrogenic NO_3_^-^/H^+^ transporter in the plasma membrane of salivary acinar cells (Qin et al., 2012), and then secreted in the oral cavity via saliva. Subsequently, some of the salivary nitrate (5%~ 36%) is reduced to nitrite by specific oral commensal bacteria in the mouth, ensuring continuous substrate delivery for oral nitrite generation (Lundberg and Govoni, 2004; Lundberg et al., 2018). Once nitrate and nitrite enter the stomach, an acid-dependent, non-enzymatic reaction converts them into bioactive nitrogen oxides and NO•, respectively (Lundberg et al., 2011).

Nitrate and nitrite have also been used as food additives in cured meats (Shakil et al., 2022).Under acidic conditions, nitrite could react with biogenic amines such as secondary or tertiary amines to form N-nitrosamines, which are potent carcinogens (Sindelar and Milkowski, 2012). Importantly, nitrate is highly stable in the body, with only a small fraction converted to nitrite, and N-nitrosamine formation requires stringent conditions. Increasing of evidence suggests no significant correlation between dietary nitrate intake and gastrointestinal tumors (van Loon et al., 1997, van Loon et al., 1998; Buller et al., 2021), while high intake of nitrates and nitrites from animal sources is associated with an increased risk of gastric cancer. In contrast, nitrate or nitrite derived from fruits and vegetables is linked to reduced gastric cancer risk (Hernández-Ramírez et al., 2009), likely due to the high antioxidant content (e.g., Ascorbic acid), which inhibit N-nitrosamine formation. The World Health Organization (WHO) recommends an upper limit of daily nitrite intake of 0.06-0.07 mg/kg (JEFCA, 1995) and a nitrate intake limit of 3.7 mg/kg for adults (Mensinga et al., 2003).

## 3 Nitrate reducase, nitrite reducase, and nitrate reduction pathways

**Figure 2:**
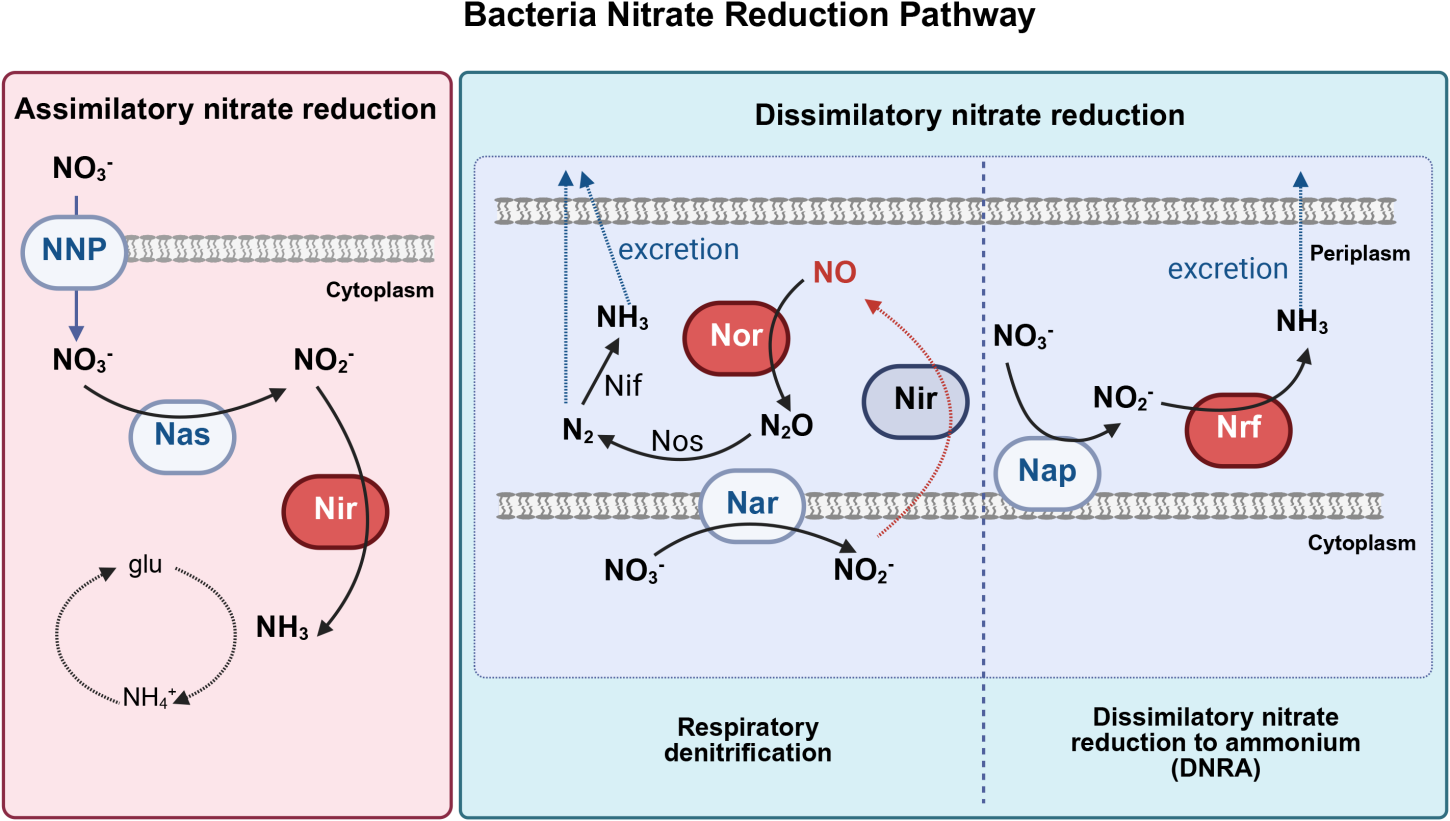
Three major bacterial nitrate reduction pathways (Adapted from Koch et al (Koch et al., 2017). and Goh et al (Goh et al., 2022), and created with BioRender.com). Bacterial nitrate reduction pathways, including assimilatory nitrate reduction, dissimilatory nitrate reduction to ammonium (DNRA), and respiratory denitrification pathways. Nas, cytoplasmic assimilatory reductases; Nar, cytoplasmic nitrate reductase; Nap, periplasmic nitrate reductase; NNP, nitrate/nitrite transporter; Nir, nitrite reductase; Nor, nitric oxide reductase; Nrf, ammonia-producing nitrite reductase, Nos, nitrous oxide reductase; Nif, nitrogenase; glu, glutamine; N_2_O, nitrous oxide; N_2_, dinitrogen; NH_3_, ammonia; NH_4_^+^, ammonium; NO_2_, nitrite; NO_3_, nitrate; NO, nitric oxide.

Numerous bacterial species possess nitrate reductase genes, which encode proteins that reduce nitrate to nitrite via molybdenum-dependent nitrate reductases. Molybdenum-dependent nitrate reductases can be classified into three major groups: periplasmic dissimilatory reductases (Nap), membrane-bound respiratory reductases (Nar), and cytoplasmic assimilatory reductases (Nas) (Koopman et al., 2016; Koch et al., 2017). Nitrate reduction can be achieved through two main pathways: assimilatory nitrate reduction (ANR) and dissimilatory nitrate reduction (DNR) (**Figure 2**) (Koch et al., 2017; Goh et al., 2022; Morou-Bermúdez et al., 2022; Rosier et al., 2022). During assimilation, nitrate is assimilated as a nitrogen source for biomass synthesis. Nitrate is reduced to nitrite via Nas in the cytoplasm and nitrite is further reduced to ammonia, which is then assimilated into the amino acid glutamine. No nitrite accumulation or ammonium release occurs during ANR. Nitrate assimilation occurs widely in bacteria, including *Methanotrophs* (e.g. *Methylobacter*, *Methylococcus*) (Ren et al., 2000), antotrophic bacteria (e.g. *Nitrosomonas, Nitrobacterm*), heterotrophic bacteria (e.g. *Enterobacteriaceae, Bacillus, Pseudomonas*) (Seenivasagan et al., 2014) which are not prevalent and abundant in oral cavity.

**Figure 3:**
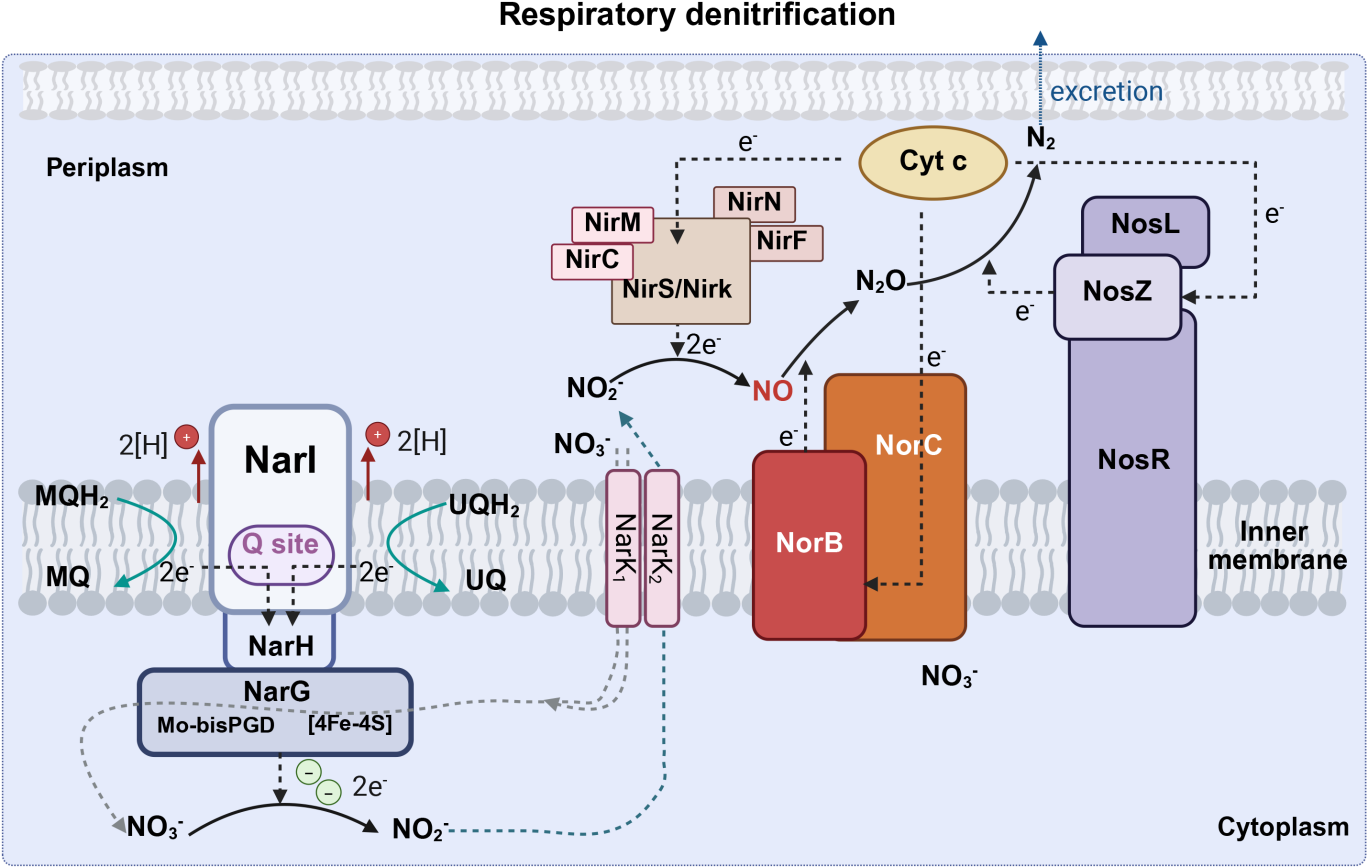
The respiratory denitrification pathway (Created with BioRender.com). Respiratory denitrification comprises a four-step reductive process in which nitrate is reduced to nitrite catalyzed by Nar, nitrite is further reduced to NO• by nitrite reductase, and then NO• is converted to N_2_O and N_2_. Nar, cytoplasmic nitrate reductase (including narG, narH, and narI); Nir, nitrite reductase (including nirC, nirF, nirK, nirM, nirN, nirS); Nor, nitric oxide reductase (including norB and norC); Nos, nitrous oxide reductase (including nosL, nosR, nosZ); Cyto C, cytochrome c; UQ, ubiquinone; UQH_2_, ubiquinol; MQ, menaquinone; MQH_2_, menaquinol; N_2_O, nitrous oxide; N_2_, dinitrogen; NH_3_, ammonia; NO_2_, nitrite; NO_3_, nitrate; NO, nitric oxide.

**Figure 4:**
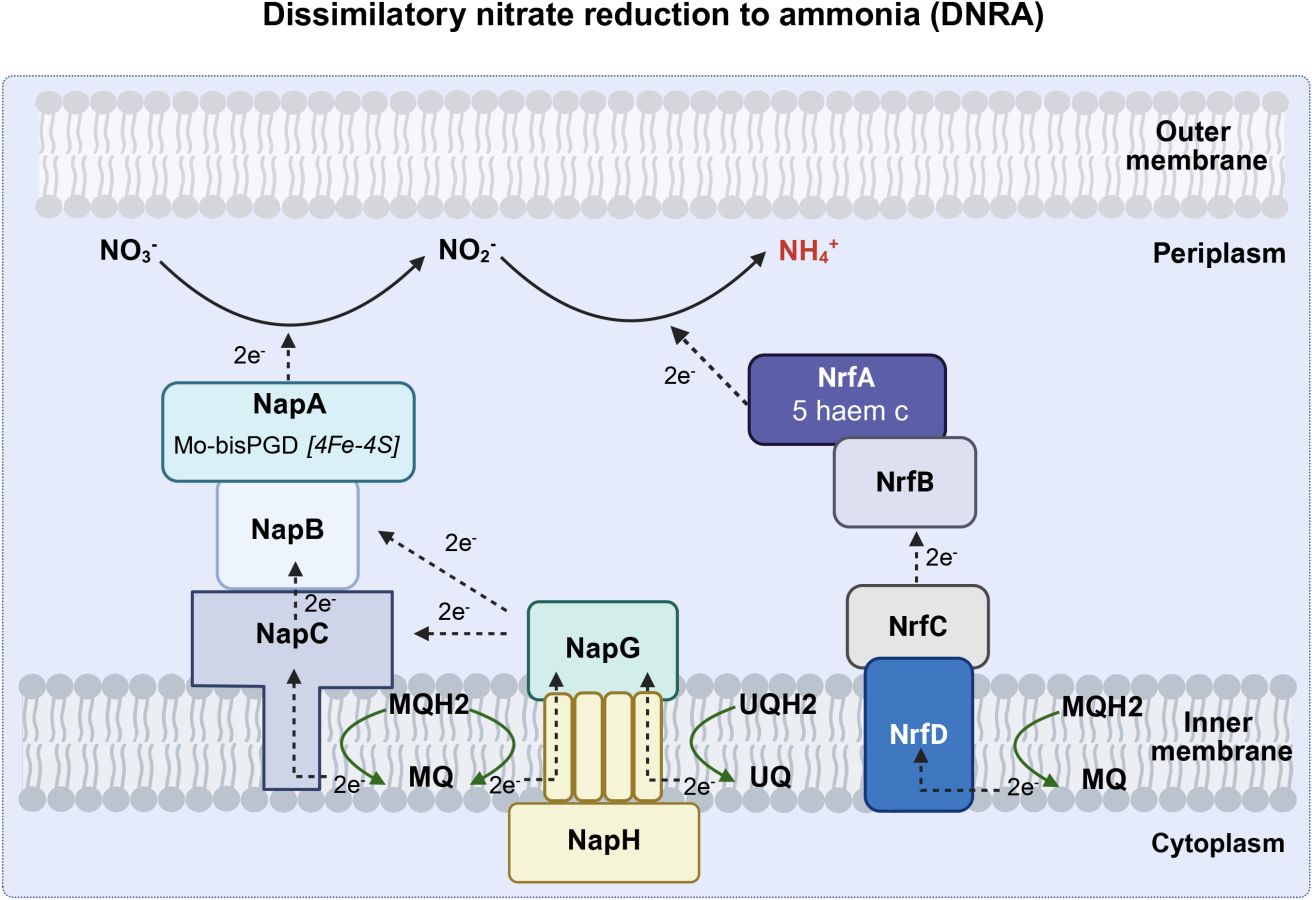
The DNRA pathway (Created with BioRender.com). Dissimilatory nitrate reduction to ammonia (DNRA) is a two-step process in which nitrate is reduced by Nap in the periplasm, converted to ammonia via an Nrf, and excreted. Nap, periplasmic nitrate reductase (including napA, napB, napC, napG and napH); Nrf, ammonia-producing nitrite reductase (including nrfA, nrfB, nrfC, nrfD), UQ, ubiquinone; UQH_2_, ubiquinol; MQ, menaquinone; MQH_2_, menaquinol; NH_4_^+^, ammonium; NO_2_, nitrite; NO_3_, nitrate; NO, nitric oxide.

DNR involves respiratory pathways in which microorganisms use NO_3_^-^ or NO_2_^-^ to replace O_2_ as an electron acceptor in respiratory metabolism under oxygen-limiting conditions (Goh et al., 2022). Respiratory denitrification comprises a four-step reductive process in which nitrate is reduced to nitrite catalyzed by Nar, nitrite is further reduced to NO• by nitrite reductase, and NO• is converted to nitrous oxide (N_2_O) and nitrogen gas(N_2_). Gaseous nitrogen can be excreted or reduced to ammonia by nitrogenase and then excreted (**Figure 3**). Dissimilatory nitrate reduction to ammonia (DNRA) is a two-step process in which nitrate is reduced by Nap in the periplasm, converted to ammonia via an ammonia-producing nitrite reductase (Nrf), and excreted (**Figure 4**).

The main bacteria responsible for DNR could use oxygen as the electron acceptor in oxygen-rich environments and nitrate as the electron acceptor in oxygen-limiting environments. Oral bacterial nitrate reduction capacity transcends traditional aerobic/anaerobic classification, as both facultative anaerobes (e.g., *Haemophilus parainfluenzae*, *Aggregatibacter actinomycetemcomitans*) and obligate aerobes (e.g., *Neisseria sicca*, *N. subflava*) harbor functional nitrate reductase systems (Rosier et al., 2022). Species of *Neisseria* (including *N.*elongata, *N.*favescens, *N.subflava, N.sicca)* possess the nitrate/nitrite reduction related genes (e.g., narG, napA, nirK, norB) (Rosier et al., 2022). *Prevotella* and *Veillonella* dominate DNRA pathways, while denitrification genes persist in aerobic-classified *Haemophilus*, and *Aggregatibacter* species. Among these bacteria, *H. parainfluenzae* and *Aggregatibacter actinomycetemcomitans* possess genes associated with denitrification and DNRA (Morou-Bermúdez et al., 2022). In summary, oral nitrate-reducing bacteria, including facultative anaerobes and obligate aerobes dynamically utilize oxygen or nitrate as electron acceptors, harboring denitrification and DNRA genetic pathways.

## 4 Oral nitrate-reducing bacteria and influencing factors

### 4.1 Oral nitrate-reducing microbiota

**Figure 5:**
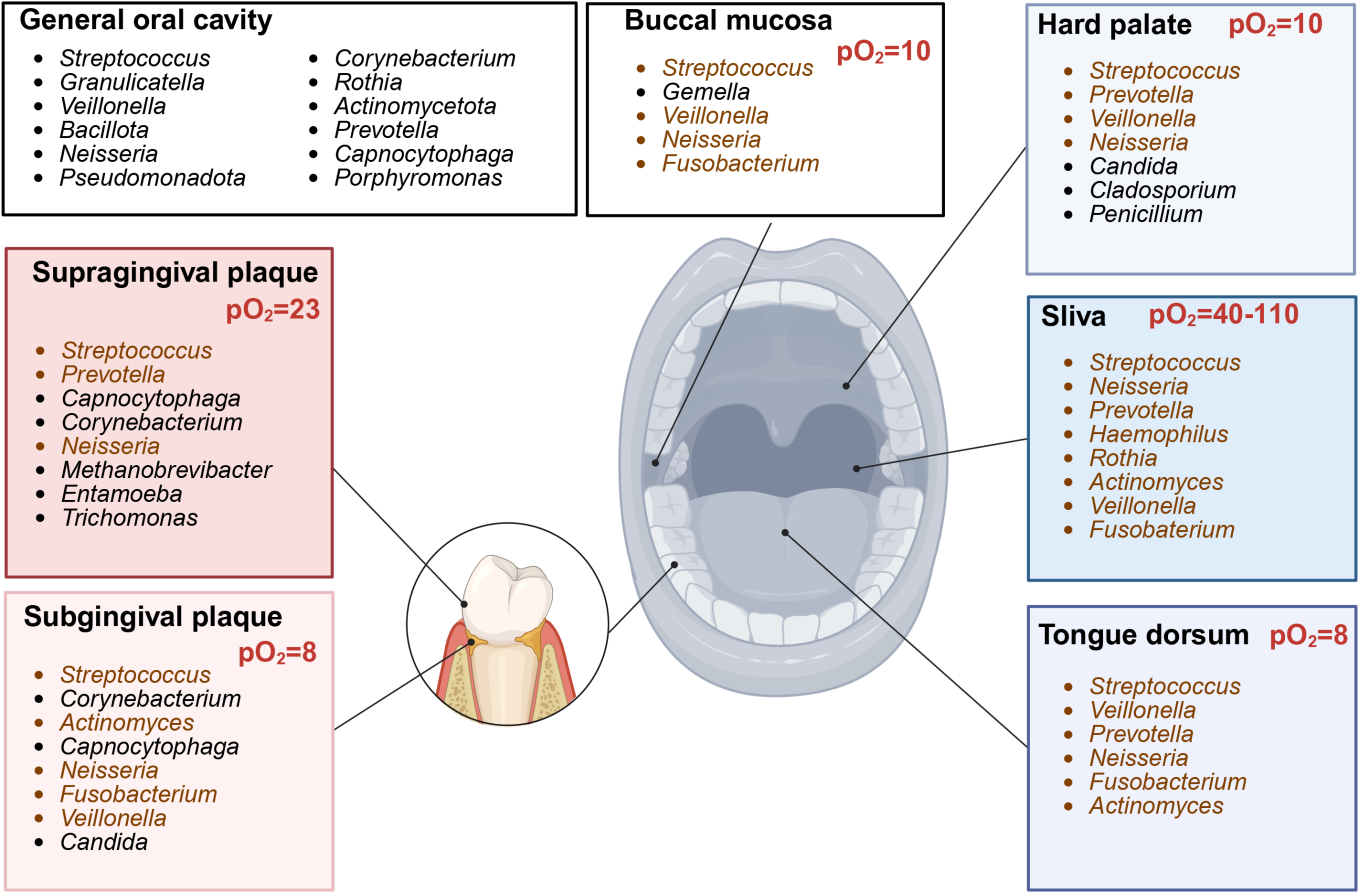
Site-specific core bacterial genera composition in oral cavity (Created with BioRender.com). Representative genera across different locations in oral cavity. Genera with nitrate-reducing ability (brown). pO2, oxygen partial pressure.

Nitrate conversion is mainly carried out in the oral cavity (Duncan et al., 1995; Lundberg and Govoni, 2004; Bryan et al., 2017). Nitrate reductase activity is the highest in the posterior one-third of the dorsum of the tongue but also occurs in the front tongue, dental plaque, and saliva under aerobic conditions (Duncan et al., 1995; Doel et al., 2005). Known oral bacteria are shown in **Figure 5**. *Veillonella*, *Neisseria*, *Haemophilus*, *Actinomyces*, *Rothia*, *Prevotella*, *Granulicatella*, *Fusobacterium*, *Staphylococcus*, and *Propionibacterium* are representative oral nitrate-reducing bacteria, identified from tongue-scraping samples (Doel et al., 2005; Huttenhower et al., 2012; Hyde et al., 2014a; Liddle et al., 2019). The most variable nitrate-reducing species are *Rothia dentocariosa* and *Haemophilus parainfluenzae*, whereas *Prevotella melaninogenica*, *Neisseria subflava*, *Rothia mucilaginosa*, *Veillonella dispa*, and *Veillonella parvula* are the most consistently abundant nitrate-reducing species (Goh et al., 2019; Liddle et al., 2019). *Staphylococcus sciuri* dominates the posterior tongue, which is the primary site of nitrite production (Li et al., 1997). Additionally, Hyde et al. identified nine other species with nitrate-reducing activity: *Granulicatella adiacens*, *Actinomyces odontolyticus*, *Actinomyces viscosus*, *Actinomyces oris*, *Neisseria flavescens*, *Neisseria mucosa*, *Neisseria sicca*, *Prevotella salivae*, and *Veillonella atypica* (**Figure 3**) (Hyde et al., 2014a).

### 4.2 Influencing factors of oral nitrate-reducing microbiota and capacity

#### 4.2.1 Sex and age

Salivary nitrate-reduction capacity exhibits age-dependent dynamics and sex differences in adults. Salivary nitrite production is undetectable or minimal in newborns, with infants exhibiting significantly lower nitrite concentrations and oral nitrate-reductase capacity compared to adults (Timby et al., 2020). Nitrate-reduction activity peaks during middle age before declining in older adults (Ahmed et al., 2021). No sex-based differences in salivary nitrate/nitrite levels have been observed at 4–12 months of age (Timby et al., 2020). In contrast, despite comparable oral microbiome structures between sexes, female adults demonstrate higher post-nitrate-supplementation nitrite levels in saliva, plasma, and urine than males (Kapil et al., 2018). This divergence may be influenced by body mass index, lifestyle factors (e.g., diet and smoking), or sex hormones—factors previously linked to NOS activity regulation (Weiner et al., 1994).

#### 4.2.2 Exogenous nitrate supplementation

Increased dietary nitrate intake, as a selective pressure for nitrate-reducing bacteria, may alter the oral microbiome, especially leading to the abundance of nitrate-reducing bacteria (Moran et al., 2024). Tongue samples of rats exhibited increased relative abundances of *Streptococcus* and *Haemophilus* (especially *H. parainfluenzae*) after nitrate supplementation (Hyde et al., 2014b). In healthy participants, dietary nitrate supplementation selectively regulated the composition of oral microbiota, characterized by a significant increase in the relative abundance of nitrate-reducing genera *Neisseria* (including *N. flavescens*, *N. subflava*) and *Rothia* (e.g., *R. mucilaginosa*), alongside a marked decrease in that of *Prevotella* (e.g., *P. melaninogenica*), *Actinomyces* (e.g., *A. hyovaginalis*), and *Veillonella* (Velmurugan et al., 2016; Vanhatalo et al., 2018; Burleigh et al., 2019; Rosier et al., 2020a; Moran et al., 2024; Zhang et al., 2025). The detailed study protocols are summarized in **Table 1**. Denitrifying species, such as *Neisseria* and *Rothia*, are associated with increased systemic NO• levels, whereas DNRA organisms such as *Prevotella* and *Veillonella* are associated with low NO• levels (Morou-Bermúdez et al., 2022). *Veillonella*, which is the most abundant nitrate-reducing genus detected in tongue scrapings (Doel et al., 2005; Bryan et al., 2017), possesses the capacity for powerful nitrate reduction. After nitrate intake, the population of *Veillonella* has been reported to decreased (**Table 1**). This discrepancy may be due to a change in oral pH (Rocha and Laranjinha, 2020).

**Table 1:** Alteration of oral bacterial communities after nitrate supplementation.

Year	Clinical study design	Sample type	Participants	Dietary nitrate intake	Main findings
2014 (Hyde et al., 2014a)	Animal study	Tongue swab	8 Wistar rats (7-week old)	Animals were supplemented with sodium nitrate in their drinking water (1 g/L) for 5 days.	Taxa that decreased in nitrate-supplemented rats include *Micrococcaceae*, *Enterobacteriaceae*, Granulicatella, and *Aggregatibacter.* The mean relative abundance of *Haemophilus* spp. and *Streptococcus* spp. increased in nitrate-supplemented rats.
2016 (Velmurugan et al., 2016)	A randomized, double-blind, placebo-controlled parallel trial	Saliva	67 nonsmoking, nondiabetic, otherwise healthy hypercholesterolemic patients	6-week period of supplementation with 250 mL beetroot juice daily or 250 mL nitrate-depleted beetroot juice.	Abundance of *Rothia mucilaginosa* and *Neisseria flavescens* increased after nitrate treatment.
2018 (Vanhatalo et al., 2018)	A randomized, double-blind, cross-over design	Tongue swab	9 old and 9 young adults healthy volunteer	2-weeks with nitrate-rich concentrated beetroot juice (2 × 70 mL/d, each 70 mL containing ~ 6.2 mmol nitrate); nitrate-depleted concentrated beetroot juice as placebo.	After nitrate supplementation the relative abundances of *Rothia* (+127%) and *Neisseria* (+351%) were greater, and *Prevotella* (-60%) and *Veillonella* (-65%) were lower than in the placebo condition;
2019 (Burleigh et al., 2019)	A placebo-controlled, single blind randomized crossover study	Tongue swab	11 healthy males	2 separate 7-day phases; participants ingested 70 ml of nitrate-rich beetroot juice (∼6.2 mmol nitrate) and nitrate-depleted beetroot juice twice per day.	1) Dietary nitrate intake reduced the relative abundance of *Prevotella*, *Streptococcus*, and *Actinomyces*. 2) The abundance of *Neisseria* increased in both groups, with a greater magnitude observed in the nitrate supplementation arm versus placebo. 3) Abundance of *N. subflava* increased, *A. hyovaginalis* and *P. melaninogenica* decreased after nitrate supplementation.
2020 (Rosier et al., 2020a)	*In vitro* study	Saliva	12 healthy volunteers	Saliva was collected to grow *in vitro* bioflms with and without 6.5 mM nitrate. Samples were taken at 5 h and 9 h of bioflm formation for 16S rRNA gene Illumina sequencing.	Significantly higher levels of *Neisseria* (3.1 ×) and *Rothia* (2.9 ×) were detected in the nitrate condition already after 5 h, while *Streptococcus, Veillonella, Oribacterium Porphyromonas, Fusobacterium, Leptotrichia, Prevotella*, and *Alloprevotella* were significantly reduced (p< 0.05 at 5 h and/or 9 h).
2024 (Black et al., 2024)	A double-blind, crossover design.	Saliva	11 healthy volunteers (10 males, 1 female)	1) A 7-day standard nitrate-diet (~180 mg nitrate/d; STD), followed by a 3-day high nitrate diet (~1000 mg nitrate/d; HIGH). 2) A 7-day low nitrate diet (~30 mg nitrate/d; LOW), followed by HIGH.	At phylum and genus levels, diminished *Proteobacteria* and *Neisseria* in LOW compared to CON; however, these P-values did not survive FDR correction.
2025 (Reichardt et al., 2025)	A double-blind, crossover design.	Saliva, tongue, and subgingival plaque	22 patients undergoing orthodontic treatment with buccal fixed appliances	Eleven subjects received daily 120 mL nitrate-containing juice for a period of 2 weeks.	1) The difference in communities based on juice consumption should be visible just after 2 weeks. 2) Nitrate consumption increased the abundances of *Neisseria* and *Abiotrophia* but decreased *Actinomyces* and *Stomatobaculum.*
2025 (Zhang et al., 2025)	A single-site experimental trial	Saliva	13 healthy subjects (8 female and 5 male, aged 18–65)	Nitrate supplements orally (concentrate from beetroot juice, 400 mg, equivalent to 6.45 mM) each morning for 5-days.	1) The abundance of nitrate-reducing bacteria increased following nitrate supplementation. 2) *Neisseria flavescens* abundance increased 1.16-fold compared to pre-supplemental levels. 3) The most abundant species observed after supplementation were *N. flavescens*, *R. mucilaginosa 1*, and *S.mitis*, accounting for 30% of the overall composition

#### 4.2.3 Mouthwash usage

Nitrite production on the tongue of adult humans is greatly reduced after administration of broad-spectrum antibacterial agents (Li et al., 1997). CHX and other antibacterial-containing mouthwashes abolish the effect of sodium nitrate supplementation (Govoni et al., 2008; Kapil et al., 2013; Pinheiro et al., 2016). CHX suppressed bacterial growth by binding and perforating cell membranes and inhibiting bacterial chemotaxis, fiagellar assembly, and lipopolysaccharide (LPS) biosynthesis and has been reported to preferentially target gram-negative bacteria because LPS is their major cell membrane component (Liu et al., 2023b). Short-term treatment with CHX decreased the relative abundance of *Prevotella*, *Fusobacterium*, and *Selenomonas* in hospitalized patients (Liu et al., 2023b). *Haemophilus* and *Aggregatibacter* were almost eliminated from the tongues of CHX-treated animals (Hyde et al., 2014b). CHX, as a potent antimicrobial, does not eradicate viable bacteria on the tongue or cause large-scale changes in the microbiome community structure, which would result in a significant reduction in bacterial viability (Tribble et al., 2019). The viability of nitrate-reducing bacteria and other conditional pathogenic bacteria decreased simultaneously after the usage of CHX or similar mouthwash products that do not target specific bacteria. Thus, it is necessary to produce a personalized antibacterial mouthwash that can effectively distinguish different functional bacteria according to different needs of patients and considering various factors.

#### 4.2.4 pH

Nitrate supplementation can increase the oral pH from 7.0 to 7.5 (Hohensinn et al., 2016), and pH 8 is optimal for nitrate reductase activity (vanMaanen et al., 1996). Nitrite can be reduced to ammonium (NH4^+^) and protons, which are consumed in the ANR and DNR pathways, resulting in an increase in the local pH. Additionally, lactic acid can act as an electron donor and carbon source in these pathways, which further modifies the pH (Rosier et al., 2022). In addition, NO• production from nitrite is pH-dependent and is increased at pH values below 5 (Rosier et al., 2022). Generally, an alkaline pH promotes DNRA over denitrification (Morou-Bermúdez et al., 2022). An acidic pH of 6 stimulates the reduction of nitrite more than that under pH 7 or pH 7.5 by the denitrification-related species *Rothia in vitro* (Rosier et al., 2020b). Furthermore, under acidic conditions, the levels of N_2_O (production of NO reduction in the respiratory denitrification pathway) are two-fold higher than those of NO•, suggesting that the reduction of NO• is also pH-dependent (Schreiber et al., 2010). Under low pH, due to the high levels of nitrate or nitrite, certain bacteria and microbial communities capable of nitrate reduction may preferentially survive or expand (Koopman et al., 2016).

#### 4.2.5 Oxygen content

Areas with high nitrate reductase activity, such as the tongue dorsum and subgingival plaque, have a low oxygen partial pressure (pO_2_, 8–13 mmHg, ~1%–2%) (Koch et al., 2017). The average oxygen concentration detected in the anterior aspect of the tongue is higher than that detected in the posterior portion (**Figure 5**) (Eskow and Loesche, 1971). The majority of oral nitrate-reducing bacteria are facultative anaerobes that prefer aerobic respiration, but they can grow under anoxic or oxygen-limiting conditions by utilizing the respiratory nitrate reductive pathway. In DNRA, a strictly anaerobic process, *Nap* expression is highest under low oxygen and nitrate conditions (Stewart, 1994). Respiratory denitrification in dental biofilms occurs under aerobic conditions (Schreiber et al., 2010); however, in an oxygen-limiting environment, *Nar* expression is only upregulated under high nitrate concentrations (Sparacino-Watkins et al., 2014). The important determining factors of bacterial respiration at specific locations are oxygen tension and nitrate concentration (Koch et al., 2017).

#### 4.2.6 Smoking

Smoking compromises oral nitrate metabolism, as demonstrated in the study of Bailey et al. (2016), who found that nitrate supplementation could not reduce blood pressure in smokers. These findings can be explained by the cyanide in cigarette smoke, which is enzymatically converted to thiocyanate, leading to elevations in circulating thiocyanate levels (serum/saliva) proportional to smoking intensity (Degiampietro et al., 1987; Bailey et al., 2016). Compared with non-smokers, the concentrations of nitrate are increased and decreased, respectively, in the plasma and saliva of smokers, while higher levels of thiocyanate exist in both plasma and saliva (Bailey et al., 2016). Thiocyanate has the potential to impede the reduction of nitrate to nitrite, or to catalyze nitrite degradation, rather than interfering with salivary nitrate re-concentration (Dewhurst-Trigg et al., 2018). Concurrently, nitrate reductase activity is suppressed by >80% in smokers, directly impairing enzymatic conversion (Ahmed et al., 2017). In addition, unstimulated salivary pH is more acidic in cigarette smokers than in non-smokers, which may have effect on nitrate reduction. Smoking reduces the overall nitrate-reducing capacity (denitrification) and aerobic taxa abundance (Antonello et al., 2023). Jia et al. collected saliva samples from 316 healthy subjects (150 subjects who had never smoked, and 166 smokers), and found that smoking significantly altered the oral microbial composition, characterized by the increased relative abundance of *Actinomyces* and *Veillonella* alongside the decreased abundance of *Neisseria* and *Haemophilus* (Jia et al., 2021). Thus, the collective impairment arises from thiocyanate, enzymatic suppression, pH alteration, and microbiota imbalance induced by smoking; however, their mechanism remains incompletely resolved.

#### 4.2.7 Periodontitis

The subgingival plaque is associated with aerobic or facultative anaerobic bacteria capable of nitrate reduction, including *Streptococcus*, *Rothia*, *Neisseria*, *Actinomyces*, and *Veillonella* (Rosier et al., 2022; Kunath et al., 2024). In dental plaque, nitrate can be converted to N_2_ via respiratory denitrification under aerobic conditions in a pH-dependent manner (Schreiber et al., 2010), and the DNRA pathway is active in anaerobic environments. Nitrite concentration is increased in the saliva and gingival crevicular fluid of patients with periodontal disease (Reher et al., 2007; Parwani et al., 2012; Sanchez et al., 2014; Topcu Ali et al., 2014). The potential mechanism underlying this phenomenon may involve iNOS activity (Batista et al., 2002; Oner et al., 2024), which is related to disease severity, and increased levels of NO• in gingival tissues and its subsequent oxidation to nitrate and nitrite.

The abundance of *Rothia* and *Neisseria*, two representative nitrate-reducing bacteria, is decreased in the subgingival plaque of patients with periodontitis (Wang et al., 2013; Kirst et al., 2015; Feres et al., 2021; Chen et al., 2022) and is negatively correlated with gingival inflammation (Huang et al., 2021; Rosier et al., 2022). Clinical studies have shown that nitrate supplementation attenuates chronic gingivitis by inhibiting gingival inflammation, resulting in an increase in the relative abundances of *Rothia* and *Neisseria* in subgingival plaque (Jockel-Schneider et al., 2016; Rosier et al., 2020a; Jockel-Schneider et al., 2021). The abundance of *Prevotella*, a major pathogen involved in periodontitis, decreases after increased nitrate intake (Vanhatalo et al., 2018; Burleigh et al., 2019). However, the relationship between nitrate-reducing bacterial abundance and periodontitis development remains unclear.

#### 4.2.8 Caries

The salivary nitrate concentration is significantly lower in patients with caries, and it is negatively correlated with the severity of caries (Zhang et al., 2021b). Typically, caries occurs at pH < 5.5, and the reduction of nitrite to NO• typically occurs under similar acidic conditions. Ammonium production during DNRA, along with lactic acid and hydrogen sulfide (as electron donors) consumption, contributes to acid prevention (Wicaksono et al., 2020; Rosier et al., 2022; Feng et al., 2023).The abundances of nitrate-reducing bacterial genera, such as *Neisseria*, *Actinomyces*, *Rothia*, *Propionibacterium*, *Haemophilus*, *Selenomonas*, and *Granulicatella*, and representative nitrate-reducing species such as *R. dentocariosa*, *Selenomonas noxia*, *Kingella oralis*, *V. dispar*, and other *Selenomonas* sp. were decreased in patients with caries (Aas et al., 2008; Tanner et al., 2011; Luo et al., 2012; Jiang et al., 2018; Xu et al., 2018; Celik et al., 2021; Yang et al., 2021). Interestingly, *Veillonella* plays an essential role in the development of caries and closely interacts with caries-associated bacteria in bacterial adhesion, co-aggregation, and biofilm formation (Feng et al., 2023). The relative abundance and prevalence of *Veillonella* are similar or higher in the oral cavity of patients with caries compared with those in the oral cavity of caries-free individuals (Tanner et al., 2011; Jiang et al., 2018; Qudeimat et al., 2021). Thus, nitrate and nitrate-reducing bacteria can prevent the development of caries by regulating the pH, reducing the accumulation of lactic acid, and increasing denitrification (Li et al., 2007; Rosier et al., 2021). Further studies are required to explore the distribution, prevalence, abundance, interactions, and effects of nitrate-reducing bacteria on caries progression.

#### 4.2.9 Salivary gland disorders

Salivary gland disorders, such as Sjogren’s syndrome (SS) or xerostomia, induce a decrease in salivary flow and acidification of the oral cavity’s pH, which affects microorganism colonization (Bustos-Lobato et al., 2023). The oral microbiome of patients with SS significantly differs from that of healthy individuals (Kim et al., 2022; Bustos-Lobato et al., 2023). Salivary gland dysfunction leads to a significant decrease in salivary nitrate concentration and an increase in urinary excretion, leading to changes in enterosalivary circulation (Xia et al., 2003a, Xia et al., 2003b). Changes in salivary nitrate levels may cause an increase in the conversion of nitrite and NO• by nitrate-inducing bacteria. Higher abundances of *Veillonella*, *Neisseria*, and *Streptococcus* have been observed in patients with SS compared with those in patients without SS (Kim et al., 2022), particularly *V. parvula* in subgingival biofilms (Singh et al., 2021). Interestingly, the abundances of other representative nitrate-reducing species, such as *H. parainfluenzae*, were significantly lower in SS than those in the controls (Tseng et al., 2021). While there has been no conclusive evidence of a link, the correlations between the nitrate-reducing microbiome composition and salivary gland dysfunction offer a potentially novel avenue for future investigations.

## 5 Nitrate-reducing microbiota in the gut

In the gut, NO• can be generated through the oxidation of L-arginine by NO synthase, and nitrate/nitrite can act as an N source for NO•. Nitrate is usually absorbed in the upper intestinal tract; approximately one third of nitrate reaches the lower intestine, and 1% is present in feces (Bartholomew and Hill, 1984). A study conducted on germ-free and normal rats showed that NO• can be produced by bacteria residing in the small intestine of normal rats but not in germ-free rats (Sobko et al., 2004). After nitrate supplementation, NO• generation in human feces is significantly increased by commensal bacteria (Sobko et al., 2005).

The gut harbors one of the largest microbial ecosystems, containing over 1 kg of bacterial biomass and up to 1,000 different species (Nicholson et al., 2005; Kunath et al., 2024). The gut microbiome mainly consists of anaerobes belonging to the phyla *Bacteroides, Firmicutes*, and *Lactobacilli*, including the genera *Bacteroides*, *Prevotella*, and *Ruminococcus* and some noticeable variations, including *Desulfovibrio* and *Akkermansia* (Cresci and Bawden, 2015; de Vos et al., 2022; Kunath et al., 2024). The major gut microbiota inhabiting differ among intestinal locations. Different healthy individuals may possess different microbiomes. Diet, stress, lifestyle, medications, local or systemic diseases, and many other factors can influence the composition of the gut microbiome.

Complex local factors in the intestinal lumen play an important role in the interaction between nitrate/nitrite and bacteria. The intestinal lumen has an estimated pO_2_ of less than 0.1 mmHg, whereas in the adjacent mucus layer, the pO_2_ is 0.1–10 mmHg (Koch et al., 2017; Rocha and Laranjinha, 2020). pO2 is highest in the proximal gut, including the gastric fundus and small bowel, and is lower in the sigmoid colon and rectum. In addition, the oxygen gradient decreases to 80–100 mmHg (~10–13%) in the submucosa and bottom of the villi to the covered mucous layer and the center of the lumen is essentially oxygen-free (Koch et al., 2017). Furthermore, the pH differs between different locations in the gut, with pH 6.37 in the ascending colon, pH 6.61 in the colon transversum, and pH 7.04 in the descending colon (Koch et al., 2017). Reducing the oxygen content and pH may influence nitrate production by gut bacteria, but limited studies have examined this.

Knowledge of the interaction between nitrate and gut microbiota remains limited. *In vitro*, nitrate is mainly reduced to ammonium via the DNRA pathway by gut microorganisms (Allison and Macfarlane, 1988; Vermeiren et al., 2009). DNRA is preferred over denitrification by gut bacteria when electron levels are limited (Vermeiren et al., 2009). The predominant nitrate reduction pathway utilized by gastrointestinal bacteria, such as *Escherichia coli*, *Lactobacillus* spp., and *Bifidobacterium* spp., or in clinical stool samples, is DNRA (Vermeiren et al., 2009; Tiso and Schechter, 2015). Therefore, nitrate is predominantly reduced to ammonium in the gut and then converted to urea in the liver (Morou-Bermúdez et al., 2022). *Escherichia coli*, *Bacteroides thetaiotaomicron*, and *Clostridium difficile* do not generate NO• via the NO_3_^–^ NO_2_^-^ -NO• pathway *in vitro*, whereas *Lactobacilli* and *Bifidobacteria* spp. generate NO from nitrite; among these species, only a few strains can generate NO from nitrate (Sobko et al., 2005). In summary, nitrate and nitrate-reducing bacteria are interconnected in the gut and play an important role in gastrointestinal and systemic health, and additional studies could further elaborate on the underlying ecological mechanisms.

## 6 Role of nitrate-reducing bacteria in systemic health

### 6.1 Cardiovascular disease

Enterosalivary nitrate plays an important role in NO• production and acts as an important mediator of the development of CVD, endothelial dysfunction, and peripheral artery diseases (Kleinbongard et al., 2006; Lundberg et al., 2015; Lundberg and Weitzberg, 2022). Oral supplementation with nitrate (such as from beetroot juice) increases circulating nitrate, nitrite and NO• levels, and blood pressure (Webb et al., 2008; Hobbs et al., 2013; Siervo et al., 2013; Liddle et al., 2019). **Table 2** presents a detailed summary of clinical studies that explored the effect of dietary nitrate intake on the CVD.

**Table 2:** Summary of clinical studies exploring the effect of dietary nitrate intake on CVD.

Year	Clinical study design	Participants	Dietary nitrate intake	Clinical parameters	Main findings
2006 (Larsen et al., 2006)	A randomized, double-blind, crossover design with two different periods during which the subjects received either nitrate or placebo.	17 healthy volunteers (15 men and 2 women; mean age, 24 years) none of whom smoked	1) 3-day sodium nitrate (0.1 mmol/kg/BW/d); 2) 3-day placebo (sodium chloride, 0.1 mmol/kg/BW/d).	1) SBP and DBP 2) Pulse rate 3) Plasma nitrate/nitrite levels	1) SBP and pulse rate did not change significantly after nitrate intake. 2) Nitrate supplementation lowered DBP (-3.7 mmHg) and mean arterial pressure (-3.2 mmHg); versus placebo. 3) Plasma nitrate/nitrite levels were significantly higher after nitrate ingestion.
2013 (Gilchrist et al., 2013)	A randomized double-blind, placebo-controlled crossover trial with two different 2-week treatment periods during which the subjects received either nitrate or placebo.	27 participants (9 women and 18 men) with T2DM	2-week period of supplementation with 250 ml beetroot juice daily or 250 ml nitrate-depleted beetroot juice.	1) Plasma nitrate and nitrite concentrations 2) 24-h ambulatory BP 3) Macro-/microvascular endothelial function	1) Median plasma nitrate/nitrite concentrations increased from 31.0 mmol/L/232 nmol/L (placebo) to 150 mmol/L/390 nmol/L (nitrate). 2) Dietary nitrate supplementation had no effect on BP or macro-/microvascular endothelial function in patients with T2DM.
2015 (Kapil et al., 2015)	A prospective single-centre, double-blind, randomized, placebo-controlled trial	68 hypertensive patients, randomization of drug-naive (n=34) and treated (n=34) hypertensive patients	4-weeks with either dietary nitrate (250 mL daily, as beetroot juice) or a placebo (250 mL daily, as nitrate-free beetroot juice).	1) Home and clinic BP 2) Vascular function, including PWV and augmentation index (AIx) 3) Transcutaneous arterial methaemoglobin concentrations	1) Clinic SBP and DBP decreased compared to baseline by 7.7 and 2.4 mmHg after nitrate intake. 2) Home SBP and DBP reduced within 1 week of consumption of dietary nitrate and reduced over the entire 4-week intervention period. 3) Compared to placebo, dietary nitrate reduced PWV by 0.58 m/s and AIx by 6.1%.
2016 (Velmurugan et al., 2016)	A randomized, double-blind, placebo-controlled parallel trial	67 nonsmoking, nondiabetic, otherwise healthy hypercholesterolemic patients	6-week period of supplementation with 250 mL beetroot juice daily or 250 mL nitrate-depleted beetroot juice.	FMD and aPWV	1) Dietary nitrate resulted in increase in the FMD response of 1.1% (an w24% improvement from baseline), and a small improvement in the aPWV (i.e., a decrease of 0.22 m/s).
2018 (Burleigh et al., 2019)	A placebo-controlled, single blind randomized crossover study	11 healthy, normotensive males (age 30 ± 7 years)	Two 7-day dietary supplementation phases 1) 70 mL beetroot juice (∼6.2 mmol nitrate) in the morning and 70 mL in the evening. 2) Same volume of nitrate-depleted beetroot juice.	1) BP and FMD 2) Salivary and plasma nitrate levels 3) 16S metagenomic sequencing of tongue swab samples	1) A transient reduction in SBP and increase in the FMD response at 2.5-hour post-nitrate supplementation. 2) Nitrate supplementation increased salivary pH (7.13 ± 0.54 to 7.39 ± 0.68). 3) Nitrate intake altered the abundance of bacteria: *Neisseria* (from 2% to–9%), *Prevotella* (from 34% to 23%) and *Actinomyces* (from 1% to 0.5%).
2018 (Vanhatalo et al., 2018)	Two 10-day dietary supplementation periods with nitrate and placebo in a randomized, double-blind, cross-over design	9 elderly adults (mean age 75 years; 6 females, 3 males); 9 young adults (mean age 20 years; 5 females, 4 males) healthy volunteer	2-weeks with nitrate-rich concentrated beetroot juice (2 × 70 mL/d, each 70 mL containing ~ 6.2 mmol nitrate); nitrate-depleted concentrated beetroot juice as placebo.	1) Plasma nitrate and nitrite 2) BP and pulse wave velocity (PWV)	Nitrate supplementation increased plasma concentration of nitrite, and reduced BP in elderly but not young subjects.
2022 (Bock et al., 2022)	Patients with T2DM completed two study visits separated by an 8-week supplementation period. A randomized, doubled-blinded, placebo-controlled parallel study.	37 patients with T2DM	1) Beetroot drink containing 250 mg nitrate (~ 4.03 mmol) and 20 mg of initrite (~0.29 mmol) 2) Placebo containing trace amounts of nitrate (5–10 mg) and no nitrite.	1) Peripheral and central BP 2) PWV, AIx	1) Nitrate/nitrite supplementation reduced peripheral SBP (148 to 142 mm Hg) but not placebo. 2) Central SBP (131 to 127 mm Hg) and augmented pressure (13.3 to 11.6 mm Hg) were reduced after nitrate/nitrite, but not placebo. 3) Peripheral and central DBP was unchanged by the interventions. 4) Nitrate/nitrite also reduced AIx (24.3% to 21.0%) whereas no changes were observed following placebo.
2023 (Rowland et al., 2024)	A repeated-measures, crossover design	12 young healthy males	1) 2 × 70 mL of concentrated nitrate-rich (13 mmol) beetroot juice; 2) nitrate-depleted (~0.04 mmol) beetroot juice.	1) urine osmolality 2) BP and pulse wave variables 3) Exercise performance determined at baseline and 2.5 h after nitrate supplement	1) Brachial SBP was unchanged following nitrate supplementation in all conditions. 2) Central SBP was reduced in every timepoint after nitrate ingestion. 3) Cycling time to exhaustion was not different between nitrate and placebo at any timepoint.
2023 (Willmott et al., 2023)	A single-site experimental trial	17 Non-pregnant normotensive (NPNT) women; 15 pregnant normotensive (PNT) women; 7 non-pregnant hypertensive (NPT) women; 12 pregnant hypertensive (PHT) women	A single dose of dietary nitrate (70 mL beetroot juice shot containing 400 mg inorganic nitrate).	Nitrate reductase (NaR) activity assays, salivary and plasma nitrate/nitrite concentrations, and BP determined at baseline and 2.5 h after nitrate supplement	1) Salivary and plasma nitrate and nitrite increased after dietary nitrate intake. 2) Nonpregnant participants had a greater decrease in SBP compared with pregnant participants and this decrease was notably greater in the NPT women.
2024 (Black et al., 2024)	A double-blind, crossover design.	11 healthy volunteers (10 males, 1 female)	1) a 7-day standard nitrate-diet (~180 mg nitrate/d; STD), followed by a 3-day high nitrate diet (~1000 mg nitrate/d; HIGH); 2) a 7-day low nitrate diet (~30 mg nitrate/d; LOW), followed by HIGH. Both interventions were preceded by 3-day STD/control diets and separated by ≥10-day washout.	1) Pulmonary gas exchange 2) BP 3) Nitrate and nitrite of saliva, plasma, and skeletal muscle	1) Following HIGH, saliva and plasma nitrate and nitrite and muscle nitrate were significantly elevated above CON, LOW and STD, but there was no difference between CON-LOW-HIGH and CON-STD-HIGH. 2) BP and exercise performance were not altered following LOW. 3) HIGH significantly reduced SBP and DBP compared to CON when preceded by STD but not when preceded by LOW. 4) Peak (+4%) and mean (+3%) power output during sprint cycling was significantly improved following HIGH.
2025 (Hayes et al., 2025)	A cross-sectional followed by a randomized, double-blind, placebo-controlled parallel study	20 postmenopausal females (60–85 yr), 10 young females	12-week period of supplementation with nitrate (8.8 mmol/day), or the same amount of placebo	1) Serum nitrate and nitrite levels 2) Carotid artery stiffness analysis	1) Nitrate supplementation significantly reduced PWVβ, β stiffness, elastic modulus, and AIx at weeks 4, 8, and 12, whereas arterial compliance increased by week 12. 2) Serum nitrate and nitrite concentrations were elevated 5- to 6 and 1.5- to 2-fold, respectively, after nitrate intake, with peak concentrations occurring at week 8. 3) Blood pressure remained unchanged in both groups.

The effects of nitrate supplementation vary across disease states. In healthy young volunteers, acute nitrate ingestion induced a transient reduction in systolic blood pressure (SBP) and an enhanced flow-mediated dilation (FMD) response at 2.5 h post-ingestion (Burleigh et al., 2018). After 3 days of supplementation, diastolic blood pressure (DBP) was observed to decrease (Larsen et al., 2006). Chronic and low dietary nitrate intake (1–2 weeks) did not have significant effects on blood pressure (BP) in young adults, whereas a reduction in BP was observed in older adults following 2 weeks of supplementation (Vanhatalo et al., 2018; Black et al., 2024). In hypertensive patients, both acute (2.5 h) and chronic (4 weeks) nitrate intake improved BP, specifically by reducing SBP, whereas no significant changes were observed in hypertensive pregnant women (Kapil et al., 2015; Willmott et al., 2023). In patients with hypertension, hypercholesterolemia, or postmenopausal women, vascular function, including pulse wave velocity (PWV), augmentation index (AIx), β stiffness and elastic modulus, demonstrated improvement following nitrate intake (Kapil et al., 2015; Velmurugan et al., 2016; Hayes et al., 2025). Similarly, dietary nitrate has been reported to prevent endothelial dysfunction, such as peripheral arterial disease (Bock et al., 2018; Hughes et al., 2022) and ischemia-reperfusion (Li et al., 2021; Zhang et al., 2021a; Yassaghi et al., 2023). Notably, individuals with type 2 diabetes mellitus (T2DM) exhibited no significant changes in BP or macro-/microvascular endothelial function following 2 weeks of nitrate supplementation (Gilchrist et al., 2013). However, extending nitrate supplementation to 8 weeks significantly reduced both peripheral and central SBP in T2DM patients, with no observed changes in DBP. Collectively, the vascular stiffening and reduced NO responsiveness in T2DM may require prolonged nitrate intervention (≥8 weeks) to achieve therapeutic effects.

The potential correlation between decreased abundance or absence of nitrate-reducing microbial communities and subsequent CVD risk remains unresolved. Epidemiological evidence suggests that oral microbial dysbiosis is linked to impaired cardiovascular health (Briskey et al., 2016). Notably, hypertensive women have significantly lower salivary nitrite concentrations and diminished relative abundance of *Veillonella* compared with those of their normotensive counterparts (Willmott et al., 2023). Furthermore, decreased oral nitrate-reducing bacterial abundance precedes the onset of preeclampsia, highlighting its potential as a predictive biomarker (Altemani et al., 2022). Collectively, these observations emphasize the essential role of oral nitrate-reducing microbiota in cardiovascular homeostasis, further supported by interventional studies demonstrating that mouthwash-induced depletion of these bacteria directly worsens cardiovascular parameters (**Table 3**). Frequent mouthwash use (≥twice daily) was associated with a higher incidence of hypertension (Joshipura et al., 2020). In healthy participants, utilization of antibacterial mouthwash resulted in elevated salivary nitrate levels and reduced nitrite levels, accompanied by transient increases in SBP and DBP within 1–4 h post-administration. There were no significant changes in BP or marginal elevations after 3 days or 1 week of use (Kapil et al., 2013; Sundqvist et al., 2016; Woessner et al., 2016; Cutler et al., 2019). For hypersensitive individuals, 3-day usage of antibacterial mouthwash resulted in a significant increase in home SBP (2.3mm Hg) (Bondonno et al., 2015). Overall, nitrate-reducing bacteria, as a critical component of the NO• generation pathway, play a pivotal role in cardiovascular regulation, and these observed associations have driven mechanistic investigations into how targeted modulation of oral nitrate-reducing microbiota alters cardiovascular outcomes.

**Table 3:** Suppression of oral nitrate-reducing microbia by antiseptic mouthwash alters cardiovascular homeostasis: Evidence from clinical trials.

Year	Participants	Clinical study design	Type of intervention	Duration of intervention (washout period)	Main findings
2013 (Kapil et al., 2013)	19 healthy individuals, aged 18–45 years; BMI 18–40 kg/m^2^; non-smokers;	A crossover study. An initial 7-day control period followed by a 7-day treatment period with CHX mouthwash	Volunteers rinsed with 10 ml 0.2% CHX mouthwash twice daily	1 week	1) Antiseptic mouthwash reduced oral nitrite production by 90% and plasma nitrite levels by 25% vs. control period. 2) SBP and DBP increased by 2–3.5 mmHg, correlated to a decrease in circulating nitrite concentrations. 3) The BP effect appeared within 1 day and was sustained during the 7-day mouthwash intervention.
2015 (Bondonno et al., 2015)	15 treated hypertensive individuals; aged 50–70 years; non-smokers; not diabetic; body mass index (BMI) 20–35kg/m^2^; SBP120-159mmHg; DBP <100 mm Hg.	A randomized controlled cross-over study. Two treatment period including mouthwash and tap water	Participants rinsed their mouths for 30 s with 20 ml of either the 1.28 mg/mol chlorhexidine gluconate antibacterial mouthwash or tap water after brushing teeth, morning and evening.	3 days (10–12 days)	1) 3-day use of antibacterial mouthwash resulted in a significant increase in home SBP (2.3 mm Hg) but did not increase DBP (0.7 mm Hg). 2) Antibacterial mouthwash significantly attenuated oral nitrate reduction capacity (nitrate reduction ratio [NR ratio]: −4.2), reduced salivary nitrite (41 vs. 111 µmol/L), and increased salivary nitrate (686 vs. 252 µmol/L), while plasma nitrate and nitrite levels remained unaffected.
2016 (Sundqvist et al., 2016)	17 healthy females; age 23 ± 4; BMI 22 ± 3	A randomized, double-blind, crossover design using an antibacterial mouthwash or lacebo	0.2% CHX mouthwash or placebo to rinse 3 times a day, 1 min each time after meals.	Two 3-day treatment periods (28 days)	1) Mouthwash elevated salivary nitrate (1118 vs. 401 μM) and reduced nitrite (23 vs. 248 μM) compared to placebo, with no significant alterations in plasma nitrate/nitrite levels. 2) 3-day use of antiseptic mouthwash did not significantly change 24 h ambulatory BP, neither during day-time or the night-time dip
2016 (Woessner et al., 2016)	12 healthy adult males	A randomized clinical trial, cross-over study.	4 mouthwash treatments consisted of: 1) water (control); 2) Listerine^®^ antiseptic mouthwash (active ingredients: Eucalyptol 0.092%, Menthol 0.042%, Methyl salicylate 0.060%, Thymol 0.064%); 3) Cepacol^®^ antibacterial mouthwash (active ingredients: Cetylpyridinium chloride 0.05%); 4) Chlorhexidine mouthwash (active ingredient: chlorhexidine gluconate 0.12%)	Participants consumed a total of 8.4 mmol nitrate. 15 min after intake, rinsed with 5 mL mouthwash solution or control for 60 s	Testing of BP at baseline and each hour for 4 h. The main effect of mouthwash treatment was significant on SBP, but not for time or mouthwash treatment time during 4 h post application of mouthwash.
2019 (Cutler et al., 2019)	23 healthy and normotensive participants	A single-site experimental trial	Participants rinsed with antibacterial mouthwash or placebo for 1 min at 1, 30, 60, and 90 min after exercise	–	Blood pressure was measured before and 1 h and 2 h after exercise. The SBP-lowering effect of exercise was attenuated by 61% at 1 h in the recovery period, and it was fully attenuated 2 h after exercise with antibacterial mouthwash.
2020 (Joshipura et al., 2020)	540 individuals; age 40–65 years; overweight/obese (BMI ≥ 25.0 kg/m^2^); not diabetic	A 3-year follow-up longitudinal cohort study	Baseline and follow-up questionnaires assessed frequency of oral hygiene aids including mouthwash use	–	1) 12% (66/540) developed hypertension over follow-up. 2) Frequent mouthwash use (≥twice daily) was associated with a higher incidence of hypertension than both infrequent use (Incidence Rate Ratio [IRR] = 1.85) and non-use (IRR = 2.17).
2025 (Bescos et al., 2025)	45 healthy individuals; aged 18–50 years; BMI < 30 kg/m^2^	A randomized clinical trial, cross-over study	Participants rinsed twice a day with 0.2% CHX or propolis mouthwash	1 week	1) A significant reduction in nitrite-producing activity (NPA) and abundance of nitrite-producing species (NPS) was observed in the CHX group compared to baseline and the propolis group. 2) At baseline, systolic and mean BP were similar, SBP and DBP were lower after CHX application without significant difference.

### 6.2 Digestive system diseases

#### 6.2.1 Stomach diseases

Nitrite and intragastric nitrogen oxides can affect physiological processes in the gastrointestinal tract, such as gastric mucosal blood flow and mucus formation (Bjorne et al., 2004; Petersson et al., 2007; Lundberg et al., 2008; Petersson et al., 2009). A small increase in intragastric NO• can be attributed to gastric or intestinal bacteria that may reduce nitrate to nitrite and NO• (Brittain et al., 1992). The acidic environment of the stomach, which has a pH between 1.5 and 3.5, is a natural barrier for most oral bacteria. Thus, only acid-resistant oral bacteria, such as *Streptococcus* spp., *Veillonella* spp., and *Prevotella* spp., are commonly found in the stomach, but their relative abundances differ (Kunath et al., 2024). *Helicobacter*, *Stenotrophomonas*, *Haemophilus*, *Streptococcus*, *Veillonella*, *Rothia, Actinomyces*, and *Prevotella* are the major genera in the stomach, as determined by pyrosequencing (Jo et al., 2016). Importantly, the pathogen *Helicobacter pylori* can neutralize gastric acidity by generating ammonium from urea using urease, enabling its survival and growth in acidic environments. *H. pylori* infection causes inflammation and alters stomach pH, ultimately reducing acidity, blocking NOS2 expression, and decreasing NO• production (Gobert and Wilson, 2016; Koch et al., 2017; Stewart et al., 2020). After co-culturing with *H. pylori*, nitrate-reducing bacteria increase the inflammation and atrophy of monocytic cells by modulating cytokine levels (Ojima et al., 2022).

Nitrate supplementation results in a 20% increase in the thickness of the firmly adherent mucus layer; this increase was absent in rats treated with antiseptic mouth spray (Petersson et al., 2009). Another study found that bilateral parotid and submandibular gland duct ligature (BPSDL) completely blocked the enterosalivary circulation of nitrate and significantly decreased the levels of gastric nitrate, nitrite, and luminal NO• in the stomach of rats. The animals in the BPSDL group displayed more severe gastric ulcers than normal rats, and nitrate administration successfully reduced the percentage of deep ulcers (Jin et al., 2013). However, the association between oral/gastrointestinal nitrate-reducing bacteria and gastric homeostasis remains unclear; therefore, the role of oral nitrate-reducing microbiota in gastric disorders warrants further investigation.

#### 6.2.2 Intestinal tract diseases

Compared with that of healthy individuals, the concentration of nitrate in the plasma of patients with gastroenteritis is high and similar to that in patients with inflammatory bowel disease (IBD) (Dykhuizen et al., 1996). Similarly, rectal NO• concentrations are significantly higher in patients with active IBD (Reinders et al., 2007) compared with that in normal controls. In a previous study, nitrite and nitrate concentrations exhibited variations that were not always in line with the disease activity index (DAI) of a dextran sodium sulfate (DSS)-induced colitis model, ranging from systemic drops to marked increases, indicating the complexity of NO• metabolism in the process of IBD (Saijo et al., 2010). *Veillonella* (including *V. parvula* and *V. dispar*), an important nitrate-reducing bacterium, is commonly enriched in the intestines of patients with IBD (Schirmer et al., 2018; Rojas-Tapias et al., 2022). Nitrate supplementation significantly alleviated epithelial cell necrosis, intestinal permeability, and disruption of tight junctions to prevent hypoxia-induced small intestinal injury (Xu et al., 2024). In aged mice, nitrate supplementation for 6 months via drinking water enhanced the integrity of the colon epithelial barrier and increased the relative abundance of some intestinal probiotics, such as *Blautia, Alloprevotella, Butyricicoccus*, and Ruminococcaceae (Wang et al., 2024). Gastrointestinal diseases are closely related to abnormal nitrate and NO metabolism, in which nitrate-reducing bacteria play an important role.

Nitrate can alter bacterial communities in the gut; however, the specific interactions between nitrate and gut bacteria remain unknown. Inorganic nitrate supplementation for 1 week or 3 weeks does not affect the gut microbial communities (Conley et al., 2017; Rocha et al., 2019). Previously, our group reported that inorganic dietary nitrate increases the abundance of *Lactobacillus* and prevents colon epithelial injury induced by total body irradiation (Wang et al., 2020). Oral nitrite supplementation prevents inflammation in DSS-induced colitis by supplying NO• (Ohtake et al., 2010). Similarly, our group previously reported that oral administration of nitrate alleviates DSS-induced colitis by regulating the microbiome in the colon by increasing the abundance of *Lactobacillus* (regulate intestinal immune response), *Ruminococcaceae_UCG-014* (related to short chain fatty acids production), and *Prevotellaceae_UCG-001* (Hu et al., 2020), suggesting that nitrate may modulate inflammatory and immune responses in IBD by reshaping the gut bacterial phenotype. Overall, these results show that the beneficial biological effects of nitrate are partially due to its ability to regulate the gut microbiome and that complex nitrate reduction in the gut microbiome needs further exploration.

### 6.3 Diabetes and other metabolic syndromes

#### 6.3.1 Diabetes

Type 2 diabetes mellitus (T2DM) results in impaired NO• bioavailability (Bahadoran et al., 2021a). Dietary nitrate supplementation reverses metabolic syndrome features (including hypertension, dyslipidemia, insulin resistance, and visceral adiposity) in aged eNOS-deficient mice (Carlström et al., 2010). Nitrate supplementation in diabetic rats/mice ameliorated glycemic parameters, including gluconeogenesis, fasting glucose, insulin, lipid profiles, and insulin resistance (Li et al., 2016; Gheibi et al., 2018; Khorasani et al., 2019). Previous animal studies have demonstrated that nitrate/nitrite reduce oxidative stress, promote adipose tissue browning, and enhance insulin secretion, thus nitrate has been used in drugs to manage diabetes (Ghasemi and Jeddi, 2017). A 12-month study of high-fat/sucrose-fed mice revealed that nitrate does not improve metabolic dysfunction and exacerbates cholesterol dysregulation, cardiac fibrosis, steatotic liver disease, and hepatocellular carcinoma progression (Sowton et al., 2025). Clinical studies have reported conflicting outcomes regarding the therapeutic efficacy of dietary nitrate in T2DM, attributed to variations in intervention duration, dosage protocols, and patient-specific vascular dysfunction, as mentioned in **Table 4**. In human clinical trials, nitrate supplementation for 4 days to 24 weeks did not improve insulin sensitivity or glycemic and lipid parameters in patients with T2DM (Gilchrist et al., 2013; Shepherd et al., 2015; Bahadoran et al., 2021b). Plasma glucose levels decreased following acute nitrate intake (Cermak et al., 2015), while exercise performance improved after chronic supplementation (Bock et al., 2022). Furthermore, a high intake of green leafy vegetables was associated with a 14% reduction in the risk of T2DM development (Carter et al., 2010). The effect and mechanism of action of nitrate in T2DM remain unknown, and the interaction of nitrate with the host microbiota may be central to the underlying mechanism (Liu et al., 2023a).

**Table 4:** Summary of clinical studies exploring the effect of dietary nitrate intake on diabetes.

Year	Clinical study design	Participants	Dietary nitrate intake	Clinical parameters	Main findings
2013 (Gilchrist et al., 2013)	A randomized double-blind, placebo-controlled crossover trial with two different 2-week treatment periods during which the subjects received either nitrate or placebo	27 participants (9 women and 18 men) with T2DM	2-week period of supplementation with 250 ml beetroot juice daily or 250 ml nitrate-depleted beetroot juice	Insulin sensitivity	Dietary nitrate supplementation did not improve insulin sensitivity in patients with T2DM. Insulin sensitivity was 5.83 ± 2.80 mg/kg/min in the placebo arm and 6.03 ± 2.56 mg/kg/min in the nitrate supplementation arm.
2015 (Cermak et al., 2015)	A double-blind crossover study, consisting of 2 test periods separated by a >14-day washout period	18 male patients with T2DM using oral glucose-lowering medication	A single bolus of NaNO3 (0.15 mmol/kg/bw) or an equimolar amount of sodium chloride (placebo)	Plasma glucose and insulin concentrations assessed every 30 mins thereafter during a 2-h period	1) Ingestion of nitrate did not attenuate the postprandial rise in plasma glucose and insulin concentrations. 2) Plasma glucose concentrations measured 2.5 h post-nitrate ingestion were significantly lower than those in the placebo group (7.5 ± 0.4 vs. 8.3 ± 0.4 mmol/L).
2015 (Shepherd et al., 2015)	A randomized, double-blind, placebo-controlled crossover trial	48 patients (35 males) with T2DM volunteers	1) 70 ml/day of beetroot juice (containing 6.43 mmol of nitrate) 2) Nitrate-depleted beetroot juice (containing 0.07 mmol of nitrate) for 4 days.	1) Treadmill walking, V&O_2_ kinetics, and heart rate 2) Six-min walk test (6MWT)	Nitrate supplementation did not alter the oxygen cost of moderate-paced walking or 6MWT performance compared to placebo.
2021 (Bahadoran et al., 2021b)	A randomized, placebo-controlled, double-blind clinical trial	64 patients with clinically diagnosed T2DM	1) 5 g/d beetroot powder (containing ~250 mg nitrate, n = 35) for 24 weeks; 2) 5 g/d placebo (containing < 25 mg nitrate, n = 29) for 24 weeks	1) Glycemic parameters including blood HbA1c, fasting serum glucose, insulin, C-peptide and lipid profiles, assessed at baseline and again at weeks 4, 12, and 24 2) Serum, urine, and saliva NO metabolites	1) No significant differences in glycemic and lipid parameters were observed between the groups over time. 2) Liver and renal function tests, as safety outcome measures, showed no undesirable changes during the study follow-up.
2022 (Bock et al., 2022)	A randomized, double-blind, placebo-controlled, 8-week trial	36 patients diagnosed with T2DM and 15 control subjects	T2DM patients consumed 1) beetroot juice containing 250 mg nitrate (4.0 mmol) and 20 mg nitrite (0.3 mmol) for 8 weeks (n=18); 2) 20 mg nitrate (~0.08 mmol) or without any nitrate (placebo) daily for 8 weeks (n = 18)	1) Plasma NO metabolites, VO_2_max and work rate capacity; 2) Skeletal muscle fiber types and oxidative capacity	1) At baseline, T2DM showed higher plasma nitrate and lower plasma nitrite levels than controls. 2) VO_2_max was lower in T2DM, as was maximal carbohydrate and fatty acid-supported oxygen consumption in permeabilized muscle fibers. 3) Nitrate/nitrite supplementation increased VO_2_max. 4) Within the nitrate/nitrite group, 42% of subjects presented improvements in both carbohydrate- and fatty acid-supported oxygen consumption in skeletal muscle.

The beneficial effects of nitrate are absent in germ-free mice, resulting in abnormal glucose tolerance and increased fat content (Cordero-Herrera et al., 2019), indicating that nitrate-reducing bacteria play important roles in the development of diabetes. In the oral cavity, nitrate-reducing bacteria are negatively associated with blood glucose levels and insulin resistance (Goh et al., 2019). DNRA activity is inversely associated with insulin resistance, fasting blood glucose, and 2-h glucose (Morou-Bermúdez et al., 2025). Specifically, a high relative abundance of *H. parainfluenzae* (nitrate-reducing bacteria) and low abundance of *N. flavescens* (nitrite-reducing bacteria) are correlated with improved insulin resistance (Bahadoran et al., 2021a). Nitrate supplementation reduces the abundance of glucose metabolism-linked genera like *Prevotella* and *Veillonella*, with *Prevotella copri* strongly associated with insulin resistance and impaired glucose tolerance (Pedersen et al., 2016; Wei et al., 2020). In a previous study of 945 overweight/obese individuals (22% of participants used mouthwash ≥ twice daily), researchers evaluated the association between mouthwash use and the development of pre-diabetes/diabetes over 3 years. Using mouthwash ≥ twice daily was associated with a significantly increased risk of pre-diabetes/diabetes (Joshipura et al., 2017). Collectively, diminished oral nitrate-reducing capacity in T2DM may exacerbate metabolic dysfunction, while nitrate supplementation may partially improve glucose homeostasis by modulating nitrate-associated microbial dysbiosis.

#### 6.3.2 Other metabolic syndromes

Nitrate has emerged as a potential therapeutic dietary supplement for obesity and related conditions, including metabolic syndrome and metabolic dysfunction-associated steatotic liver disease (MASLD). Dietary nitrate can prevent metabolic syndrome and liver steatosis induced by a high-fat diet (HFD) (Liu et al., 2021). An HFD-induces hyperlipidemia and insulin resistance in mice, but these are alleviated by dietary nitrate supplementation (Li et al., 2016). Dietary nitrate attenuated HFD-induced pathological features, including developed increased myocardial fibrosis, glucose intolerance, and adipose inflammation, in HFD-fed mice (Petrick et al., 2023). Moreover, an HFD can alter intestinal microbial community composition (Petrick et al., 2023) and the bioavailability of oxygen and nitrate to gut bacteria (Yoo et al., 2021). Our group found that nitrate, nitrite, and cGMP levels increased after nitrate loading, and the abundances of *Bacteroidales S24–7* and *Alistipes* were increased in an obesity model (Ma et al., 2020). These findings demonstrate the central role of the microbiome in the bioactivation of nitrate in metabolic syndromes; however, the specific influence of nitrate-reducing bacteria on metabolic activity requires further study.

### 6.4 Brain diseases

The function of the NO_3_^–^ NO_2_^-^ -NO• pathway is associated with cognitive function, cerebral blood flow, and improvements in Alzheimer’s disease (AD) and Parkinson’s Disease (PD) (Alharbi et al., 2023; Boulares et al., 2025; Tripodi et al., 2025). Dietary nitrate has been reported to improve neurobehavioral function in mice after traumatic brain injury (Liu et al., 2025) and ameliorates myelin loss in mice with AD (Chen et al., 2025). In 63 individuals with alcohol use disorder exhibiting varying levels of cognitive impairment, reductions in the relative abundance of nitrate-reducing bacteria were correlated with more severe cognitive deficits. In mice with chronic alcohol exposure, nitrate supplementation ameliorated cognitive dysfunction and attenuated oral microbiota dysbiosis (Li et al., 2025). Nitrate/nitrite supplementation improves cognitive performance outcomes in healthy middle-aged and older humans (Justice et al., 2015; Vanhatalo et al., 2021), improves regional brain perfusion (Presley et al., 2011) and modulates the cerebral blood-flow (CBF) response to task performance (Wightman et al., 2015), as shown in **Table 5**. Furthermore, given that hypertension is a modifiable risk factor for AD, any agent that results in elevated BP could potentially increase the risk of developing this neurodegenerative disease.

**Table 5:** Summary of clinical studies exploring the effect of dietary nitrate intake on brain diseases.

Year	Clinical study design	Participants	Dietary nitrate intake	Clinical parameters	Main findings
2011 (Presley et al., 2011)	A double-blind, placebo-controlled, crossover study	16 individuals with an age cutoff of ≥ 70 years old	High nitrate diet and low nitrate diet three times daily with a wash-out period of 24 h	1) Cerebral blood flow (CBF) was determined from MR images; 2) Perfusion imaging preprocessing	1) There was no significant difference in the average global CBF between low nitrate diet (43 ± 10 ml/100 g/min) and high nitrate die (44 ± 10 ml/100 g/min). 2) Nitrate increased regional cerebral perfusion in frontal lobe white matter, especially between the dorsolateral prefrontal cortex and anterior cingulate cortex.
2015 (Wightman et al., 2015)	A double-blind, placebo-controlled, crossover study	40 healthy adults	1) 450 ml organic beetroot juice (containing 5.5 mmol nitrate) b) A placebo drink with negligible nitrate	1) Functional near-infrared spectroscopy (NIRS) 2) Plasma nitrite levels 3) Cognitive tasks after 90 min following nitrate ingestion	1) Dietary nitrate modulated the hemodynamic response, with an initial increase in CBF at the start of the task period, followed by consistent reductions. 2) Cognitive performance was improved after nitrate ingestion.
2020 (Fan et al., 2020)	A single-center, placebo-controlled, single-blinded, randomized, parallel group clinical trial	30 patients diagnosed with an acute transient ischemic attack (TIA)	1) Sodium nitrate (10 mg/kg/day) 2) Placebo for 7 days	1) Cardiorespiratory parameters: BP, pulse pressure (PP), augmentation index (AI), and reflected wave transit time (RWTT) 2) Cerebrovascular parameter: middle cerebral artery blood velocity (MCAv), total (THb)-, oxy(O_2_ Hb)-, and deoxyhemoglobin (HHb) and cerebral cortical tissue O_2_saturation (ScO_2_) 3) Cerebrovascular CO_2_ Reactivity Test 4) Blood pressure variabilit (BPV) dynamic cerebral autoregulation (CA)	1) High- and low-frequency BP-MCAv gain and MCAv-CO_2_ slope increased 7 days following TIA onset, while low-frequency BPV decreased compared with baseline. 2) Dietary nitrate elevated plasma nitrate concentration by ~547% and significantly lowered BPV (d=0.6), MCAv variability (d = 0.7), and BP-MCAv coherence (d = 0.7) in the very-low-frequency range (0.02– 0.07 Hz) 3) MCAv-CO_2_ slope and arterial stiffness were unaffected after nitrate supplementation
2022 (Fan et al., 2020)	A randomized, single-blind, placebo-controlled, four-arm parallel feasibility trial.	62 subjects with a BMI range between 25 and 40 kg/m^2^	1) High nitrate: two 70 mL shots of beetroot juice/d (approximately ~400 mg/shot), one every morning (~08:00) and one every evening (21:00) (n = 16) 2) Medium nitrate: one shot of beetroot juice every evening (21:00) (n=17). 3. Low nitrate: one shot of concentrated beetroot juice every other evening (~21:00) (n = 14). 4. Placebo: one shot of nitrate-depleted beetroot juice (~0.001 mg) every other evening (21:00) (n = 15) for 13 weeks	1) Cognitive Function 2) Quantitative NIRS	Cognitive function and CBF were not affected by supplementation with nitrate for 13 weeks.
2025 (Rajendra et al., 2025)	A cohort study	Participants were cognitively unimpaired individuals who had β-amyloid positron emission tomography (PET) scans (n = 554) and magnetic resonance imaging (MRI) scans (n = 335)	Intake of dietary nitrate from different sources where nitrate is naturally present and is an allowed additive was assessed using the food frequency questionnaire and quantified in grams/day (g/d)	1) Cerebral aβ positron emission tomography 2) Magnetic resonance imaging 3) absence of the apolipoprotein E (APOE) ϵ4 allele	1) In women with the APOE ϵ4 allele, higher plant-sourced nitrate intake (median intake 121 mg/day) was associated with a slower rate of cerebral aβ deposition [β: 4.47 versus 8.99/18 months] and right hippocampal atrophy [-0.01 versus -0.03 mm^3^/18 months, p < 0.01]. 2) Moderate intake showed protective associations in men carriers and in both men and women noncarriers of APOE ϵ4.
2025 (Pedrinolla et al., 2025)	A placebo-controlled, randomized, double-blind crossover study	1) 10 individuals with AD (76 ± 9 years), 2) 10 healthy elderly (OLD, 75 ± 6 years); 3) 10 young individuals (YN, 25 ± 4 years)	1) A single dose of nitrate-rich beetroot juice (containing 5 mmol, or 400 mg of nitrate) 2) A nitrate-depleted PLA	1) Plasmatic nitrate and nitrite kinetics 2) Vascular responsiveness via single passive limb movement (PLM) after 4 h following nitrate ingestion	1) Plasma nitrate and nitrite increased significantly in all three groups after 1 h and remained elevated for the rest of the trial. 2) Patients with AD exhibited significantly lower ΔPLM values at any time point compared to YN and OLD. 3) The same trend was found in ΔPLM, which significantly increased in all three groups over time.

Oral microbiome alterations are associated with AD severity, and gut bacterial communities are closely related to the progress of AD, although the role of nitrate, NO• and nitrate-reducing bacteria in the development of AD remains unknown (Boulares et al., 2025). The abundances of salivary *Neisseria* and *Haemophilus*, which have recently been found to be associated with improved cognitive function in older adults, increase following dietary nitrate intake (Vanhatalo et al., 2021). The bioavailability of NO• has been recognized as a risk factor for AD, and depletion of NO• is related to cardiovascular and central nervous system degenerative processes in patients with AD (Venturelli et al., 2018). However, in a recent study, Pedrinolla et al. found that patients with AD were able to reduce nitrate to nitrite and increase NO-mediated vascular responsiveness to the levels observed in healthy volunteers (Pedrinolla et al., 2025). The effect of bioavailability of NO• on AD requires further research, and targeting nitrate-reducing bacteria in patients with AD is a promising future clinical research direction.

In addition, nitrate-containing compounds have been identified as common headache triggers (Sun-Edelstein and Mauskop, 2009). In oral samples, nitrate, nitrite, and nitric oxide reductase gene expression is significantly higher in patients with migraine. In addition, there are small but significant increases in nitrate, nitrite, and nitric oxide reductase gene expression in stool samples have been collected from migraineurs. The significantly different dominant oral bacterial species between patients with and without migraines belong to the genera *Streptococcus* and *Pseudomonas*, both of which have the potential to reduce nitrate concentrations (Gonzalez et al., 2016).

## 7 Futures research directions

Nitrate reduction-related bacteria are widely distributed in the oral cavity and gut and play vital roles in the systemic circulation and bioactivation of NO. Specific bacterial strains that possess nitrate and nitrite reductases have been shown to be involved in the reduction of nitrate and nitrite. Existing research has extensively characterized oral nitrate-reducing bacteria, identifying key genera such as *Rothia, Neisseria, Veillonella*, and *Prevotella*. However, exogenous nitrate supplementation elicits divergent shifts. The relative abundance of *Rothia* and *Neisseria* significantly increased, whereas that of *Veillonella* and *Prevotella* decreased. The mechanisms underlying these compositional changes remain unclear and warrant further investigation. In addition, oral pathologies, including periodontitis and dental caries, alter the abundance of nitrate-reducing bacteria. Notably, periodontitis is closely linked to systemic diseases (Genco and Sanz, 2020), yet the role of nitrate-reducing microbiota in this oral-systemic axis remains underexplored.

Simultaneously, nitrate regulates the oral and gut microbiomes, which synergistically enhances the biofunction of nitrate. Multiple systemic or local diseases are partially caused by bacterial imbalances, and nitrate has been reported to effectively regulate bacterial abundances. Studies on intestinal nitrate-reducing bacteria are limited. For instance, *Veillonella* contributes to IBD (Rojas-Tapias et al., 2022), but the functional roles of other nitrate reducers in the gut remain poorly defined. The use of high-throughput sequencing techniques and bioinformatics technology has been increasingly used to understand the roles of bacteria with nitrate reductase activity, especially in gut. With synergistic enhancement of our understanding of the microbiome, the clinical application value of nitrates could be significantly improved.

Nitrate-derived nitrite and NO, which are reduced by these bacteria, benefit the cardiovascular system, as evidenced by clinical studies. Targeting nitrate metabolism and nitrate-reducing microbiota represents a promising therapeutic strategy for CVD. While animal models highlight the significance of nitrate and nitrate-reducing bacteria in metabolic syndrome and neurocognitive disorders, clinical evidence remains inconsistent or limited, necessitating further human clinical trials.

Notably, CHX mouthwash indiscriminately eliminates oral bacteria (including nitrate reducing bacteria) and increases BP. Thus, designing selective antimicrobial agents that target pathogenic bacteria while preserving nitrate-reducing taxa could optimize oral and systemic health. In addition, targeted mouthwashes containing nitrate-reducing agents or NO donors may offer a novel approach to personalize oral health management, showing their efficacy in modulating blood pressure and systemic NO levels.

## 8 Summary

This review systematically investigated enterosalivary nitrate metabolism, delineated nitrate reduction pathways in the oral and gut microbiomes, and analyzed the influencing factors of nitrate-reducing bacteria. We evaluated evidence linking these microbial communities to systemic diseases, particularly CVD, gastrointestinal diseases, metabolic syndromes, and brain disorders. While the causal relationships are incompletely characterized, emerging clinical data suggest that depletion of oral nitrate-reducing microbiota exacerbates cardiovascular pathogenesis and may elevate risks for developing other systemic diseases.

This review highlights that dietary nitrate alleviates systemic dysfunction through the enterosalivary nitrate circulation. Dysbiosis of nitrate-reducing bacteria is correlated with CVD, obesity, T2DM, IBD, AD, and other systemic disorders. Thus, elucidating mechanisms underlying oral-gut nitrate-reducing microbiota dysbiosis may provide foundational insights for improving human health. Targeted modulation of nitrate metabolism and nitrate-reducing communities across the oral-gut axis could serve as protective strategies against systemic diseases, emphasizing the importance of oral health maintenance. Probiotics and dietary interventions targeting these microbial consortia may be promising therapeutic avenues.

## References

[B1] Aas J. A. Griffen A. L. Dardis S. R. Lee A. M. Olsen I. Dewhirst F. E.. (2008). Bacteria of dental caries in primary and permanent teeth in children and young adults. J. Clin. Microbiol. 46, 1407–1417. doi: 10.1128/jcm.01410-07 18216213 PMC2292933

[B2] Ahmed K. A. Kim K. Ricart K. van der Pol W. Qi X. Bamman M. M.. (2021). Potential role for age as a modulator of oral nitrate reductase activity. Nitric. Oxide 108, 1–7. doi: 10.1016/j.niox.2020.12.001 33321206 PMC8085911

[B3] Ahmed K. A. Nichols A. L. Honavar J. Dransfield M. T. Matalon S. Patel R. P. (2017). Measuring nitrate reductase activity from human and rodent tongues. Nitric. Oxide 66, 62–70. doi: 10.1016/j.niox.2017.04.001 28390999 PMC5484083

[B4] Alderton W. K. Cooper C. E. Knowles R. G. (2001). Nitric oxide synthases: Structure, function and inhibition. Biochem. J. 357, 593–615. doi: 10.1042/0264-6021:3570593 11463332 PMC1221991

[B5] Alharbi M. Stephan B. C. Shannon O. M. Siervo M. (2023). Does dietary nitrate boost the effects of caloric restriction on brain health? Potential physiological mechanisms and implications for future research. Nutr. Metab. (Lond) 20, 45. doi: 10.1186/s12986-023-00766-9 37880786 PMC10599060

[B6] Allison C. Macfarlane G. T. (1988). Effect of nitrate on methane production and fermentation by slurries of human faecal bacteria. J. Gen. Microbiol. 134, 1397–1405. doi: 10.1099/00221287-134-6-1397 3221192

[B7] Altemani F. Barrett H. L. Callaway L. K. McIntyre H. D. Dekker Nitert M. (2022). Reduced abundance of nitrate-reducing bacteria in the oral microbiota of women with future preeclampsia. Nutrients 14, 1139. doi: 10.3390/nu14061139 35334796 PMC8953404

[B8] Antonello G. Blostein F. Bhaumik D. Davis E. Gögele M. Melotti R.. (2023). Smoking and salivary microbiota: A cross-sectional analysis of an italian alpine population. Sci. Rep. 13, 18904.doi.org/10.1038/s41598–023-42474-7. doi: 10.1038/s41598-023-42474-7 37919319 PMC10622503

[B9] Archer D. L. (2002). Evidence that ingested nitrate and nitrite are beneficial to health. J. Food Prot 65, 872–875. doi: 10.4315/0362-028X-65.5.872 12030305

[B10] Babateen A. M. Fornelli G. Donini L. M. Mathers J. C. Siervo M. (2018). Assessment of dietary nitrate intake in humans: A systematic review. Am. J. Clin. Nutr. 108, 878–888. doi: 10.1093/ajcn/nqy108 30321271

[B11] Bahadoran Z. Mirmiran P. Carlström M. Ghasemi A. (2021a). Inorganic nitrate: A potential prebiotic for oral microbiota dysbiosis associated with type 2 diabetes. Nitric. Oxide 116, 38–46. doi: 10.1016/j.niox.2021.09.001 34506950

[B12] Bahadoran Z. Norouzirad R. Mirmiran P. Gaeini Z. Jeddi S. Shokri M.. (2021b). Effect of inorganic nitrate on metabolic parameters in patients with type 2 diabetes: A 24-week randomized double-blind placebo-controlled clinical trial. Nitric. Oxide 107, 58–65. doi: 10.1016/j.niox.2020.12.005 33340674

[B13] Bahra M. Kapil V. Pearl V. Ghosh S. Ahluwalia A. (2012). Inorganic nitrate ingestion improves vascular compliance but does not alter flow-mediated dilatation in healthy volunteers. Nitric. Oxide-Biology Chem. 26, 197–202. doi: 10.1016/j.niox.2012.01.004 PMC3405527 22285857

[B14] Bailey S. J. Blackwell J. R. Wylie L. J. Holland T. Winyard P. G. Jones A. M. (2016). Improvement in blood pressure after short-term inorganic nitrate supplementation is attenuated in cigarette smokers compared to non-smoking controls. Nitric. Oxide 61, 29–37. doi: 10.1016/j.niox.2016.10.002 27744007

[B15] Bailey S. J. Winyard P. Vanhatalo A. Blackwell J. R. Dimenna F. J. Wilkerson D. P.. (2009). Dietary nitrate supplementation reduces the o2 cost of low-intensity exercise and enhances tolerance to high-intensity exercise in humans. J. Appl. Physiol. (1985) 107, 1144–1155. doi: 10.1152/japplphysiol.00722.2009 19661447

[B16] Bartholomew B. Hill M. J. (1984). The pharmacology of dietary nitrate and the origin of urinary nitrate. Food Chem. Toxicol. 22, 789–795. doi: 10.1016/0278-6915(84)90116-9 6541617

[B17] Batista A. C. Silva T. A. Chun J. H. Lara V. S. (2002). Nitric oxide synthesis and severity of human periodontal disease. Oral. Dis. 8, 254–260. doi: 10.1034/j.1601-0825.2002.02852.x 12363110

[B18] Bescos R. du Toit L. Redondo-Rio A. Warburton P. J. Nicholas T. L. Kiernan M.. (2025). The comparative effect of propolis and chlorhexidine mouthwash on oral nitrite-producing bacteria and blood pressure regulation. J. Oral. Microbiol. 17, 2439636. doi: 10.1080/20002297.2024.2439636 39691165 PMC11650436

[B19] Bjorne H. Petersson J. Phillipson M. Weltzberg E. Holm L. Lundberg J. O. (2004). Nitrite in saliva increases gastric mucosal blood flow and mucus thickness. J. Clin. Invest. 113, 106–114. doi: 10.1172/JCI200419019 14702114 PMC300767

[B20] Black M. I. Wylie L. J. Kadach S. Piknova B. Park J. W. Stoyanov Z.. (2024). Effects of low and high dietary nitrate intake on human saliva, plasma and skeletal muscle nitrate and nitrite concentrations and their functional consequences. Free Radic. Biol. Med. 225, 881–893. doi: 10.1016/j.freeradbiomed.2024.10.282 39401733

[B21] Bock J. M. Hughes W. E. Ueda K. Feider A. J. Hanada S. Casey D. P. (2022). Dietary inorganic nitrate/nitrite supplementation reduces central and peripheral blood pressure in patients with type 2 diabetes mellitus. Am. J. Hypertens. 35, 803–809. doi: 10.1093/ajh/hpac068 35639721 PMC9434218

[B22] Bock J. M. Treichler D. P. Norton S. L. Ueda K. Hughes W. E. Casey D. P. (2018). Inorganic nitrate supplementation enhances functional capacity and lower-limb microvascular reactivity in patients with peripheral artery disease. Nitric. Oxide 80, 45–51. doi: 10.1016/j.niox.2018.08.007 30118808 PMC6239203

[B23] Bondonno C. P. Liu A. H. Croft K. D. Considine M. J. Puddey I. B. Woodman R. J.. (2015). Antibacterial mouthwash blunts oral nitrate reduction and increases blood pressure in treated hypertensive men and women. Am. J. Hypertens. 28, 572–575. doi: 10.1093/ajh/hpu192 25359409

[B24] Boulares A. Jdidi H. Bragazzi N. L. (2025). Impact of mouthwash-induced oral microbiome disruption on alzheimer’s disease risk: A perspective review. Int. Dent. J. 75, 45–50. doi: 10.1016/j.identj.2024.07.005 39379282 PMC11806309

[B25] Briskey D. Tucker P. S. Johnson D. W. Coombes J. S. (2016). Microbiota and the nitrogen cycle: Implications in the development and progression of cvd and ckd. Nitric. Oxide-Biology Chem. 57, 64–70. doi: 10.1016/j.niox.2016.05.002 27164294

[B26] Brittain T. Blackmore R. Greenwood C. Thomson A. J. (1992). Bacterial nitrite-reducing enzymes. Eur. J. Biochem. 209, 793–802. doi: 10.1111/j.1432-1033.1992.tb17350.x 1425687

[B27] Bryan N. S. Tribble G. Angelov N. (2017). Oral microbiome and nitric oxide: The missing link in the management of blood pressure. Curr. Hypertension Rep. 19, 33. doi: 10.1007/s11906-017-0725-2 28353075

[B28] Buller I. D. Patel D. M. Weyer P. J. Prizment A. Jones R. R. Ward M. H. (2021). Ingestion of nitrate and nitrite and risk of stomach and other digestive system cancers in the iowa women’s health study. Int. J. Environ. Res. Public Health 18. doi: 10.3390/ijerph18136822 PMC8297261 34202037

[B29] Burleigh M. C. Liddle L. Monaghan C. Muggeridge D. J. Sculthorpe N. Butcher J. P.. (2018). Salivary nitrite production is elevated in individuals with a higher abundance of oral nitrate-reducing bacteria. Free Radic. Biol. Med. 120, 80–88. doi: 10.1016/j.freeradbiomed.2018.03.023 29550328

[B30] Burleigh M. Liddle L. Muggeridge D. J. Monaghan C. Sculthorpe N. Butcher J.. (2019). Dietary nitrate supplementation alters the oral microbiome but does not improve the vascular responses to an acute nitrate dose. Nitric. Oxide-Biology Chem. 89, 54–63. doi: 10.1016/j.niox.2019.04.010 31051259

[B31] Bustos-Lobato L. Rus M. J. Saúco C. Simon-Soro A. (2023). Oral microbial biomap in the drought environment: Sjogren’s syndrome. Mol. Oral. Microbiol. 38, 400–407. doi: 10.1111/omi.12435 37767604

[B32] Carlström M. Larsen F. J. Nyström T. Hezel M. Borniquel S. Weitzberg E.. (2010). Dietary inorganic nitrate reverses features of metabolic syndrome in endothelial nitric oxide synthase-deficient mice. Proc. Natl. Acad. Sci. U.S.A. 107, 17716–17720. doi: 10.1073/pnas.1008872107 20876122 PMC2955084

[B33] Carter P. Gray L. J. Troughton J. Khunti K. Davies M. J. (2010). Fruit and vegeta ble intake and incidence of type 2 diabetes mellitus: Systematic review and meta-analysis. BMJ 341, c4229. doi: 10.1136/bmj.c4229 20724400 PMC2924474

[B34] Celik Z. C. Cakiris A. Abaci N. Yaniikoglu F. Ilgin C. Ekmekci S. S.. (2021). The complex microbiome of caries-active and caries-free supragingival plaques in permanent dentition. Niger J. Clin. Pract. 24, 1535–1540. doi: 10.4103/njcp.njcp_49_21 34657022

[B35] Cermak N. M. Hansen D. Kouw I. W. van Dijk J. W. Blackwell J. R. Jones A. M.. (2015). A single dose of sodium nitrate does not improve oral glucose tolerance in patients with type 2 diabetes mellitus. Nutr. Res. 35, 674–680. doi: 10.1016/j.nutres.2015.05.017 26092495

[B36] Chen X. Hu G. Chang L. Li X. Tang Y. Wu Y.. (2025). Nitrate ameliorates myelin loss and cognitive impairment in alzheimer’s disease through upregulation of neuronal sialin and subsequent inhibition of tppp phosphorylation. Sci. Bull. (Beijing) 70, 1224–1229. doi: 10.1016/j.scib.2025.03.017 40102090

[B37] Chen T. Marsh P. D. Al-Hebshi N. N. (2022). Smdi: An index for measuring subgingival microbial dysbiosis. J. Dent. Res. 101, 331–338. doi: 10.1177/00220345211035775 34428955 PMC8982011

[B38] Coggan A. R. Leibowitz J. L. Spearie C. A. Kadkhodayan A. Thomas D. P. Ramamurthy S.. (2015). Acute dietary nitrate intake improves muscle contractile function in patients with heart failure: A double-blind, placebo-controlled, randomized trial. Circ. Heart Fail 8, 914–920. doi: 10.1161/circheartfailure.115.002141 26179185 PMC4573847

[B39] Conley M. N. Roberts C. Sharpton T. J. Iwaniec U. T. Hord N. G. (2017). Increasing dietary nitrate has no effect on cancellous bone loss or fecal microbiome in ovariectomized rats. Mol. Nutr. Food Res. 61, 1600372. doi: 10.1002/mnfr.201600372 28087899 PMC5434898

[B40] Cordero-Herrera I. Kozyra M. Zhuge Z. McCann Haworth S. Moretti C. Peleli M.. (2019). Amp-activated protein kinase activation and nadph oxidase inhibition by inorganic nitrate and nitrite prevent liver steatosis. Proc. Natl. Acad. Sci. U.S.A. 116, 217–226. doi: 10.1073/pnas.1809406115 30559212 PMC6320503

[B41] Cresci G. A. Bawden E. (2015). Gut microbiome: What we do and don’t know. Nutr. Clin. Pract. 30, 734–746. doi: 10.1177/0884533615609899 26449893 PMC4838018

[B42] Cutler C. Kiernan M. Willis J. R. Gallardo-Alfaro L. Casas-Agustench P. White D.. (2019). Post-exercise hypotension and skeletal muscle oxygenation is regulated by nitrate-reducing activity of oral bacteria. Free Radic. Biol. Med. 143, 252–259. doi: 10.1016/j.freeradbiomed.2019.07.035 31369841

[B43] D’El-Rei J. Cunha A. R. Trindade M. Neves M. F. (2016). Beneficial effects of dietary nitrate on endothelial function and blood pressure levels. Int. J. Hypertens. 2016, 6791519. doi: 10.1155/2016/6791519 27088010 PMC4819099

[B44] Degiampietro P. Peheim E. Drew D. Graf H. Colombo J. P. (1987). Determination of thiocyanate in plasma and saliva without deproteinisation and its validation as a smoking parameter. J. Clin. Chem. Clin. Biochem. 25, 711–717. doi: 10.1515/cclm.1987.25.10.711 3694128

[B45] DeMartino A. W. Kim-Shapiro D. B. Patel R. P. Gladwin M. T. (2019). Nitrite and nitrate chemical biology and signalling. Br. J. Pharmacol. 176, 228–245. doi: 10.1111/bph.14484 30152056 PMC6295445

[B46] de Vos W. M. Tilg H. Van Hul M. Cani P. D. (2022). Gut microbiome and health: Mechanistic insights. Gut 71, 1020–1032. doi: 10.1136/gutjnl-2021-326789 35105664 PMC8995832

[B47] Dewhurst-Trigg R. Yeates T. Blackwell J. R. Thompson C. Linoby A. Morgan P. T.. (2018). Lowering of blood pressure after nitrate-rich vegeta ble consumption is abolished with the co-ingestion of thiocyanate-rich vegeta bles in healthy normotensive males. Nitric. Oxide 74, 39–46. doi: 10.1016/j.niox.2018.01.009 29360600

[B48] Doel J. J. Benjamin N. Hector M. P. Rogers M. Allaker R. P. (2005). Evaluation of bacterial nitrate reduction in the human oral cavity. Eur. J. Oral. Sci. 113, 14–19. doi: 10.1111/j.1600-0722.2004.00184.x 15693824

[B49] Duncan C. Dougall H. Johnston P. Green S. Brogan R. Leifert C.. (1995). Chemical generation of nitric-oxide in the mouth from the enterosalivary circulation of ditery nitrate. Nat. Med. 1, 546–551. doi: 10.1038/nm0695-546 7585121

[B50] Dykhuizen R. S. Masson J. McKnight G. Mowat A. N. Smith C. C. Smith L. M.. (1996). Plasma nitrate concentration in infective gastroenteritis and inflammatory bowel disease. Gut 39, 393–395. doi: 10.1136/gut.39.3.393 8949643 PMC1383345

[B51] Eskow R. N. Loesche W. J. (1971). Oxygen tensions in the human oral cavity. Arch. Oral. Biol. 16, 1127–1128. doi: 10.1016/0003-9969(71)90218-4 5293413

[B52] Fan J. L. O’Donnell T. Lanford J. Croft K. Watson E. Smyth D.. (2020). Dietary nitrate reduces blood pressure and cerebral artery velocity fluctuations and improves cerebral autoregulation in transient ischemic attack patients. J. Appl. Physiol. (1985) 129, 547–557. doi: 10.1152/japplphysiol.00160.2020 32758038

[B53] Feng J. Liu J. Jiang M. Chen Q. Zhang Y. Yang M.. (2023). The role of oral nitrate-reducing bacteria in the prevention of caries: A review related to caries and nitrate metabolism. Caries Res. 57, 119–132. doi: 10.1159/000529162 36649690

[B54] Feres M. Retamal-Valdes B. Gonçalves C. Cristina Figueiredo L. Teles F. (2021). Did omics change periodontal therapy? Periodontol 85, 182–209. doi: 10.1111/prd.12358 33226695

[B55] Forstermann U. Sessa W. C. (2012). Nitric oxide synthases: Regulation and function. Eur. Heart J. 33, 829. doi: 10.1093/eurheartj/ehr304 21890489 PMC3345541

[B56] Gee L. C. Ahluwalia A. (2016). Dietary nitrate lowers blood pressure: Epidemiological, pre-clinical experimental and clinical trial evidence. Curr. Hypertens. Rep. 18, 17. doi: 10.1007/s11906-015-0623-4 26815004 PMC4729801

[B57] Genco R. J. Sanz M. (2020). Clinical and public health implications of periodontal and systemic diseases: An overview. . Periodontol 83, 7–13. doi: 10.1111/prd.12344 32385880

[B58] Ghasemi A. Jeddi S. (2017). Anti-obesity and anti-diabetic effects of nitrate and nitrite. Nitric. Oxide 70, 9–24. doi: 10.1016/j.niox.2017.08.003 28804022

[B59] Gheibi S. Jeddi S. Carlström M. Gholami H. Ghasemi A. (2018). Effects of long-term nitrate supplementation on carbohydrate metabolism, lipid profiles, oxidative stress, and inflammation in male obese type 2 diabetic rats. Nitric. Oxide 75, 27–41.doi.org/10.1016/j.niox.2018.02.002. doi: 10.1016/j.niox.2018.02.002 29432804

[B60] Gilchrist M. Winyard P. G. Aizawa K. Anning C. Shore A. Benjamin N. (2013). Effect of dietary nitrate on blood pressure, endothelial function, and insulin sensitivity in type 2 diabetes. Free Radic. Biol. Med. 60, 89–97. doi: 10.1016/j.freeradbiomed.2013.01.024 23395779

[B61] Gobert A. P. Wilson K. T. (2016). The immune battle against helicobacter pylori infection: No offense. Trends Microbiol. 24, 366–376. doi: 10.1016/j.tim.2016.02.005 26916789 PMC4841705

[B62] Goh C. E. Bohn B. Marotz C. Molinsky R. Roy S. Paster B. J.. (2022). Nitrite generating and depleting capacity of the oral microbiome and cardiometabolic risk: Results from origins. J. Am. Heart Assoc. 11, e023038. doi: 10.1161/jaha.121.023038 35574962 PMC9238569

[B63] Goh C. E. Trinh P. Colombo P. C. Genkinger J. M. Mathema B. Uhlemann A. C.. (2019). Association between nitrate-reducing oral bacteria and cardiometabolic outcomes: Results from origins. J. Am. Heart Assoc. 8, e013324. doi: 10.1161/jaha.119.013324 31766976 PMC6912959

[B64] Gonzalez A. Hyde E. Sangwan N. Gilbert J. A. Viirre E. Knight R. (2016). Migraines are correlated with higher levels of nitrate-, nitrite-, and nitric oxide-reducing oral microbes in the american gut project cohort. Msystems 1, e00105–16. doi: 10.1128/mSystems.00105-16 27822557 PMC5080405

[B65] Govoni M. Jansson E. A. Weitzberg E. Lundberg J. O. (2008). The increase in plasma nitrite after a dietary nitrate load is markedly attenuated by an antibacterial mouthwash. Nitric. Oxide-Biology Chem. 19, 333–337. doi: 10.1016/j.niox.2008.08.003 18793740

[B66] Hayes E. Alhulaefi S. Siervo M. Whyte E. Kimble R. Matu J.. (2025). Inter-individual differences in the blood pressure lowering effects of dietary nitrate: A randomised double-blind placebo-controlled replicate crossover trial. Eur. J. Nutr. 64, 101. doi: 10.1007/s00394-025-03616-x 39992469 PMC11850510

[B67] Hernández-Ramírez R. U. Galván-Portillo M. V. Ward M. H. Agudo A. González C. A. Oñate-Ocaña L. F.. (2009). Dietary intake of polyphenols, nitrate and nitrite and gastric cancer risk in Mexico city. Int. J. Cancer 125, 1424–1430. doi: 10.1002/ijc.24454 19449378 PMC2787087

[B68] Hobbs D. A. George T. W. Lovegrove J. A. (2013). The effects of dietary nitrate on blood pressure and endothelial function: A review of human intervention studies. Nutr. Res. Rev. 26, 210–222.doi.org/10.1017/s0954422413000188. doi: 10.1017/S0954422413000188 24134873

[B69] Hohensinn B. Haselgrubler R. Muller U. Stadlbauer V. Lanzerstorfer P. Lirk G.. (2016). Sustaining elevated levels of nitrite in the oral cavity through consumption of nitrate-rich beetroot juice in young healthy adults reduces salivary ph. Nitric. Oxide-Biology Chem. 60, 10–15. doi: 10.1016/j.niox.2016.08.006 27593618

[B70] Hu L. Jin L. Xia D. Zhang Q. Ma L. Zheng H.. (2020). Nitrate ameliorates dextran sodium sulfate-induced colitis by regulating the homeostasis of the intestinal microbiota. Free Radic. Biol. Med. 152, 609–621. doi: 10.1016/j.freeradbiomed.2019.12.002 31811920

[B71] Huang S. He T. Yue F. Xu X. Wang L. Zhu P.. (2021). Longitudinal multi-omics and microbiome meta-analysis identify an asymptomatic gingival state that links gingivitis, periodontitis, and aging. mBio 12, e03281–20. doi: 10.1128/mBio.03281-20 33688007 PMC8092283

[B72] Hughes W. E. Treichler D. P. Ueda K. Bock J. M. Casey D. P. (2022). Sodium nitrate supplementation improves blood pressure reactivity in patients with peripheral artery disease. Nutr. Metab. Cardiovasc. Dis. 32, 710–714. doi: 10.1016/j.numecd.2021.12.002 35090799 PMC8857030

[B73] Huttenhower C. Gevers D. Knight R. Abubucker S. Badger J. H. Chinwalla A. T.. (2012). Structure, function and diversity of the healthy human microbiome. Nature 486, 207–214. doi: 10.1038/nature11234 22699609 PMC3564958

[B74] Hyde E. R. Andrade F. Vaksman Z. Parthasarathy K. Jiang H. Parthasarathy D. K.. (2014a). Metagenomic analysis of nitrate-reducing bacteria in the oral cavity: Implications for nitric oxide homeostasis. PloS One 9, e88645. doi: 10.1371/journal.pone.0088645 24670812 PMC3966736

[B75] Hyde E. R. Luk B. Cron S. Kusic L. McCue T. Bauch T.. (2014b). Characterization of the rat oral microbiome and the effects of dietary nitrate. Free Radic. Biol. Med. 77, 249–257. doi: 10.1016/j.freeradbiomed.2014.09.017 25305639

[B76] Jia Y. J. Liao Y. He Y. Q. Zheng M. Q. Tong X. T. Xue W. Q.. (2021). Association between oral microbiota and cigarette smoking in the chinese population. Front. Cell Infect. Microbiol. 11. doi: 10.3389/fcimb.2021.658203 PMC8195269 34123872

[B77] Jiang Q. Liu J. Chen L. Gan N. Yang D. (2018). The oral microbiome in the elderly with dental caries and health. Front. Cell Infect. Microbiol. 8. doi: 10.3389/fcimb.2018.00442 PMC6328972 30662876

[B78] Jin L. Y. Qin L. Z. Xia D. S. Liu X. B. Fan Z. P. Zhang C. M.. (2013). Active secretion and protective effect of salivary nitrate against stress in human volunteers and rats. Free Radic. Biol. Med. 57, 61–67. doi: 10.1016/j.freeradbiomed.2012.12.015 23277147 PMC4059197

[B79] Jo H. J. Kim J. Kim N. Park J. H. Nam R. H. Seok Y. J.. (2016). Analysis of gastric microbiota by pyrosequencing: Minor role of bacteria other than helicobacter pylori in the gastric carcinogenesis. Helicobacter 21, 364–374. doi: 10.1111/hel.12293 26915731

[B80] Jockel-Schneider Y. Goßner S. K. Petersen N. Stölzel P. Hägele F. Schweiggert R. M.. (2016). Stimulation of the nitrate-nitrite-no-metabolism by repeated lettuce juice consumption decreases gingival inflammation in periodontal recall patients: A randomized, double-blinded, placebo-controlled clinical trial. J. Clin. Periodontol 43, 603–608. doi: 10.1111/jcpe.12542 26969836

[B81] Jockel-Schneider Y. Schlagenhauf U. Stölzel P. Goßner S. Carle R. Ehmke B.. (2021). Nitrate-rich diet alters the composition of the oral microbiota in periodontal recall patients. J. Periodontol 92, 1536–1545. doi: 10.1002/jper.20-0778 33742692

[B82] Jones A. M. Vanhatalo A. Seals D. R. Rossman M. J. Piknova B. Jonvik K. L. (2021). Dietary nitrate and nitric oxide metabolism: Mouth, circulation, skeletal muscle, and exercise performance. Med. Sci. Sports Exerc 53, 280–294. doi: 10.1249/mss.0000000000002470 32735111

[B83] Joshipura K. Muñoz-Torres F. Fernández-Santiago J. Patel R. P. Lopez-Candales A. (2020). Over-the-counter mouthwash use, nitric oxide and hypertension risk. Blood Press 29, 103–112. doi: 10.1080/08037051.2019.1680270 31709856 PMC7125030

[B84] Joshipura K. J. Muñoz-Torres F. J. Morou-Bermudez E. Patel R. P. (2017). Over-the-counter mouthwash use and risk of pre-diabetes/diabetes. Nitric. Oxide 71, 14–20. doi: 10.1016/j.niox.2017.09.004 28939409 PMC6628144

[B85] Justice J. N. Johnson L. C. DeVan A. E. Cruickshank-Quinn C. Reisdorph N. Bassett C. J.. (2015). Improved motor and cognitive performance with sodium nitrite supplementation is related to small metabolite signatures: A pilot trial in middle-aged and older adults. Aging (Albany NY) 7, 1004–1021. doi: 10.18632/aging.100842 26626856 PMC4694069

[B86] Kapil V. Haydar S. M. A. Pearl V. Lundberg J. O. Weitzberg E. Ahluwalia A. (2013). Physiological role for nitrate-reducing oral bacteria in blood pressure control. Free Radic. Biol. Med. 55, 93–100. doi: 10.1016/j.freeradbiomed.2012.11.013 23183324 PMC3605573

[B87] Kapil V. Khambata R. S. Robertson A. Caulfield M. J. Ahluwalia A. (2015). Dietary nitrate provides sustained blood pressure lowering in hypertensive patients: A randomized, phase 2, double-blind, placebo-controlled study. Hypertension 65, 320–327. doi: 10.1161/hypertensionaha.114.04675 25421976 PMC4288952

[B88] Kapil V. Rathod K. S. Khambata R. S. Bahra M. Velmurugan S. Purba A.. (2018). Sex differences in the nitrate-nitrite-no center dot pathway: Role of oral nitrate-reducing bacteria. Free Radic. Biol. Med. 126, 113–121. doi: 10.1016/j.freeradbiomed.2018.07.010 30031863

[B89] Khorasani V. Jeddi S. Yaghmaei P. Tohidi M. Ghasemi A. (2019). Effect of long-term sodium nitrate administration on diabetes-induced anemia and glucose homeostasis in obese type 2 diabetic male rats. Nitric. Oxide 86, 21–30. doi: 10.1016/j.niox.2019.02.003 30772502

[B90] Kim D. Jeong Y. J. Lee Y. Choi J. Park Y. M. Kwon O. C.. (2022). Correlation between salivary microbiome of parotid glands and clinical features in primary sjögren’s syndrome and non-sjögren’s sicca subjects. Front. Immunol. 13. doi: 10.3389/fimmu.2022.874285 PMC9114876 35603219

[B91] Kirst M. E. Li E. C. Alfant B. Chi Y. Y. Walker C. Magnusson I.. (2015). Dysbiosis and alterations in predicted functions of the subgingival microbiome in chronic periodontitis. Appl. Environ. Microbiol. 81, 783–793. doi: 10.1128/aem.02712-14 25398868 PMC4277562

[B92] Kleinbongard P. Dejam A. Lauer T. Jax T. Kerber S. Gharini P.. (2006). Plasma nitrite concentrations reflect the degree of endothelial dysfunction in humans. Free Radic. Biol. Med. 40, 295–302. doi: 10.1016/j.freeradbiomed.2005.08.025 16413411

[B93] Knowles R. G. Moncada S. (1994). Nitric-oxide synthases in mammals. Biochem. J. 298, 249–258. doi: 10.1042/bj2980249 7510950 PMC1137932

[B94] Koch C. D. Gladwin M. T. Freeman B. A. Lundberg J. O. Weitzberg E. Morris A. (2017). Enterosalivary nitrate metabolism and the microbiome: Intersection of microbial metabolism, nitric oxide and diet in cardiac and pulmonary vascular health. Free Radic. Biol. Med. 105, 48–67. doi: 10.1016/j.freeradbiomed.2016.12.015 27989792 PMC5401802

[B95] Koopman J. E. Buijs M. J. Brandt B. W. Keijser B. J. Crielaard W. Zaura E. (2016). Nitrate and the origin of saliva influence composition and short chain fatty acid production of oral microcosms. Microb. Ecol. 72, 479–492. doi: 10.1007/s00248-016-0775-z 27155967 PMC4937104

[B96] Kunath B. J. De Rudder C. Laczny C. C. Letellier E. Wilmes P. (2024). The oral-gut microbiome axis in health and disease. Nat. Rev. Microbiol. 22, 791–805. doi: 10.1038/s41579-024-01075-5 39039286

[B97] Larsen F. J. Ekblom B. Sahlin K. Lundberg J. O. Weitzberg E. (2006). Effects of dietary nitrate on blood pressure in healthy volunteers. N Engl. J. Med. 355, 2792–2793. doi: 10.1056/NEJMc062800 17192551

[B98] Li H. Duncan C. Townend J. Killham K. Smith L. M. Johnston P.. (1997). Nitrate-reducing bacteria on rat tongues. Appl. Environ. Microbiol. 63, 924–930. doi: 10.1128/AEM.63.3.924-930.1997 9055411 PMC168385

[B99] Li S. Jin H. Sun G. Zhang C. Wang J. Xu H.. (2021). Dietary inorganic nitrate protects hepatic ischemia-reperfusion injury through nrf2-mediated antioxidative stress. Front. Pharmacol. 12. doi: 10.3389/fphar.2021.634115 PMC8215696 34163351

[B100] Li T. Lu X. Sun Y. Yang X. (2016). Effects of spinach nitrate on insulin resistance, endothelial dysfunction markers and inflammation in mice with high-fat and high-fructose consumption. Food Nutr. Res. 60, 32010. doi: 10.3402/fnr.v60.32010 27616738 PMC5018658

[B101] Li X. Ni Z. Shi W. Zhao K. Zhang Y. Liu L.. (2025). Nitrate ameliorates alcohol-induced cognitive impairment via oral microbiota. J. Neuroinflamm. 22, 106. doi: 10.1186/s12974-025-03439-x PMC12001487 40234914

[B102] Li H. Thompson I. Carter P. Whiteley A. Bailey M. Leifert C.. (2007). Salivary nitrate–an ecological factor in reducing oral acidity. Oral. Microbiol. Immunol. 22, 67–71. doi: 10.1111/j.1399-302X.2007.00313.x 17241173

[B103] Liddle L. Burleigh M. C. Monaghan C. Muggeridge D. J. Sculthorpe N. Pedlar C. R.. (2019). Variability in nitrate-reducing oral bacteria and nitric oxide metabolites in biological fluids following dietary nitrate administration: An assessment of the critical difference. Nitric. Oxide 83, 1–10. doi: 10.1016/j.niox.2018.12.003 30528912

[B104] Liu T. Chen Y. C. Jeng S. L. Chang J. J. Wang J. Y. Lin C. H.. (2023b). Short-term effects of chlorhexidine mouthwash and listerine on oral microbiome in hospitalized patients. Front. Cell Infect. Microbiol. 13. doi: 10.3389/fcimb.2023.1056534 PMC9932516 36816590

[B105] Liu Y. Croft K. D. Caparros-Martin J. O’Gara F. Mori T. A. Ward N. C. (2021). Beneficial effects of inorganic nitrate in non-alcoholic fatty liver disease. Arch. Biochem. Biophys. 711, 109032. doi: 10.1016/j.abb.2021.109032 34520731

[B106] Liu Z. Hu L. Wang Q. Zhao X. Liu W. Zhang B.. (2025). Inorganic nitrate attenuates neuroinflammation after traumatic brain injury via sialin. Tissue Cell 96, 102955. doi: 10.1016/j.tice.2025.102955 40373612

[B107] Liu H. Huang Y. Huang M. Wang M. Ming Y. Chen W.. (2023a). From nitrate to no: Potential effects of nitrate-reducing bacteria on systemic health and disease. Eur. J. Med. Res. 28, 425. doi: 10.1186/s40001-023-01413-y 37821966 PMC10566198

[B108] Lundberg J. O. Carlström M. Larsen F. J. Weitzberg E. (2011). Roles of dietary inorganic nitrate in cardiovascular health and disease. Cardiovasc. Res. 89, 525–532. doi: 10.1093/cvr/cvq325 20937740

[B109] Lundberg J. O. Carlström M. Weitzberg E. (2018). Metabolic effects of dietary nitrate in health and disease. Cell Metab. 28, 9–22. doi: 10.1016/j.cmet.2018.06.007 29972800

[B110] Lundberg J. O. Gladwin M. T. Weitzberg E. (2015). Strategies to increase nitric oxide signalling in cardiovascular disease. Nat. Rev. Drug Discov. 14, 623–641. doi: 10.1038/nrd4623 26265312

[B111] Lundberg J. O. Govoni M. (2004). Inorganic nitrate is a possible source for systemic generation of nitric oxide. Free Radic. Biol. Med. 37, 395–400. doi: 10.1016/j.freeradbiomed.2004.04.027 15223073

[B112] Lundberg J. O. Weitzberg E. (2022). Nitric oxide signaling in health and disease. Cell 185, 2853–2878. doi: 10.1016/j.cell.2022.06.010 35931019

[B113] Lundberg J. O. Weitzberg E. Gladwin M. T. (2008). The nitrate-nitrite-nitric oxide pathway in physiology and therapeutics. Nat. Rev. Drug Discov. 7, 156–167. doi: 10.1038/nrd2466 18167491

[B114] Luo A. H. Yang D. Q. Xin B. C. Paster B. J. Qin J. (2012). Microbial profiles in saliva from children with and without caries in mixed dentition. Oral. Dis. 18, 595–601. doi: 10.1111/j.1601-0825.2012.01915.x 22458262

[B115] Ma L. S. Hu L. Feng X. Y. Wang S. L. (2018). Nitrate and nitrite in health and disease. Aging Dis. 9, 938–945. doi: 10.14336/ad.2017.1207 30271668 PMC6147587

[B116] Ma L. Hu L. Jin L. Wang J. Li X. Wang W.. (2020). Rebalancing glucolipid metabolism and gut microbiome dysbiosis by nitrate-dependent alleviation of high-fat diet-induced obesity. BMJ Open Diabetes Res. Care 8, e1001255. doi: 10.1136/bmjdrc-2020-001255 PMC7449567 32843498

[B117] Mensinga T. T. Speijers G. J. Meulenbelt J. (2003). Health implications of exposure to environmental nitrogenous compounds. Toxicol Rev 22, 41–51. doi: 10.2165/00139709-200322010-00005 14579546

[B118] Moran S. P. Rosier B. T. Henriquez F. L. Burleigh M. C. (2024). The effects of nitrate on the oral microbiome: A systematic review investigating prebiotic potential. J. Oral. Microbiol. 16, 2322228. doi: 10.1080/20002297.2024.2322228 38420038 PMC10901185

[B119] Morou-Bermúdez E. Guo K. Morales Morales J. Ricart K. Patel R. P. Clemente J. C.. (2025). Nitrate reduction by salivary bacteria, glucose metabolism, and lifestyle. J. Oral. Microbiol. 17, 2489612. doi: 10.1080/20002297.2025.2489612 40224947 PMC11986870

[B120] Morou-Bermúdez E. Torres-Colón J. E. Bermúdez N. S. Patel R. P. Joshipura K. J. (2022). Pathways linking oral bacteria, nitric oxide metabolism, and health. J. Dent. Res. 101, 623–631. doi: 10.1177/00220345211064571 35081826 PMC9124908

[B121] Munzel T. Daiber A. (2018). Inorganic nitrite and nitrate in cardiovascular therapy: A better alternative to organic nitrates as nitric oxide donors? Vascul Pharmacol. 102, 1–10. doi: 10.1016/j.vph.2017.11.003 29174923

[B122] Nicholson J. K. Holmes E. Wilson I. D. (2005). Gut microorganisms, mammalian metabolism and personalized health care. Nat. Rev. Microbiol. 3, 431–438. doi: 10.1038/nrmicro1152 15821725

[B123] Ohtake K. Koga M. Uchida H. Sonoda K. Ito J. Uchida M.. (2010). Oral nitrite ameliorates dextran sulfate sodium-induced acute experimental colitis in mice. Nitric. Oxide 23, 65–73. doi: 10.1016/j.niox.2010.04.004 20399279

[B124] Ojima H. Kuraoka S. Okanoue S. Okada H. Gotoh K. Matsushita O.. (2022). Effects of helicobacter pylori and nitrate-reducing bacteria coculture on cells. Microorganisms 10, 2495. doi: 10.3390/microorganisms10122495 36557748 PMC9785519

[B125] Oliveira-Paula G. H. Pinheiro L. C. Tanus-Santos J. E. (2019). Mechanisms impairing blood pressure responses to nitrite and nitrate. Nitric. Oxide-Biology Chem. 85, 35–43. doi: 10.1016/j.niox.2019.01.015 30716418

[B126] Oner F. Onat F. C. Ozkan Karasu Y. (2024). Salivary and serum nitric oxide synthase, macrophage inflammatory protein 1 alpha and macrophage migration inhibitory factor levels in periodontal disease. Heliyon 10, e25888. doi: 10.1016/j.heliyon.2024.e25888 38384515 PMC10878917

[B127] Parwani S. R. Chitnis P. J. Parwani R. N. (2012). Salivary nitric oxide levels in inflammatory periodontal disease - a case-control and interventional study. Int. J. Dent. Hyg 10, 67–73. doi: 10.1111/j.1601-5037.2011.00508.x 21564536

[B128] Pedersen H. K. Gudmundsdottir V. Nielsen H. B. Hyotylainen T. Nielsen T. Jensen B. A.. (2016). Human gut microbes impact host serum metabolome and insulin sensitivity. Nature 535, 376–381. doi: 10.1038/nature18646 27409811

[B129] Pedrinolla A. Dorelli G. Porcelli S. Burleigh M. Mendo M. Martignon C.. (2025). Increasing nitric oxide availability via ingestion of nitrate-rich beetroot juice improves vascular responsiveness in individuals with alzheimer’s disease. Nitric. Oxide 156, 50–56. doi: 10.1016/j.niox.2025.03.001 40089052

[B130] Petersson J. Carlstrom M. Schreiber O. Phillipson M. Christoffersson G. Jagare A.. (2009). Gastroprotective and blood pressure lowering effects of dietary nitrate are abolished by an antiseptic mouthwash. Free Radic. Biol. Med. 46, 1068–1075. doi: 10.1016/j.freeradbiomed.2009.01.011 19439233

[B131] Petersson J. Jadert C. Phillipson M. Borniquel S. Lundberg J. O. Holm L. (2015). Physiological recycling of endogenous nitrate by oral bacteria regulates gastric mucus thickness. Free Radic. Biol. Med. 89, 241–247. doi: 10.1016/j.freeradbiomed.2015.07.003 26163002

[B132] Petersson J. Phillipson M. Jansson E. A. Patzak A. Lundberg J. O. Holm L. (2007). Dietary nitrate increases gastric mucosal blood flow and mucosal defense. Am. J. Physiology-Gastrointestinal Liver Physiol. 292, G718–G724. doi: 10.1152/ajpgi.00435.2006 17082222

[B133] Petrick H. L. Ogilvie L. M. Brunetta H. S. Robinson A. Kirsh A. J. Barbeau P. A.. (2023). Dietary nitrate and corresponding gut microbiota prevent cardiac dysfunction in obese mice. Diabetes 72, 844–856. doi: 10.2337/db22-0575 36812497

[B134] Piknova B. Park J. W. Kwan Jeff Lam K. Schechter A. N. (2016). Nitrate as a source of nitrite and nitric oxide during exercise hyperemia in rat skeletal muscle. Nitric. Oxide 55-56, 55–56. doi: 10.1016/j.niox.2016.03.005 PMC4860042 27000467

[B135] Piknova B. Park J. W. Swanson K. M. Dey S. Noguchi C. T. Schechter A. N. (2015). Skeletal muscle as an endogenous nitrate reservoir. Nitric. Oxide 47, 10–16. doi: 10.1016/j.niox.2015.02.145 25727730 PMC4439352

[B136] Pinheiro L. C. Ferreira G. C. Amaral J. H. Portella R. L. Tella S. D. C. Passos M. A.. (2016). Oral nitrite circumvents antiseptic mouthwash-induced disruption of enterosalivary circuit of nitrate and promotes nitrosation and blood pressure lowering effect. Free Radic. Biol. Med. 101, 226–235. doi: 10.1016/j.freeradbiomed.2016.10.013 27769921

[B137] Presley T. D. Morgan A. R. Bechtold E. Clodfelter W. Dove R. W. Jennings J. M.. (2011). Acute effect of a high nitrate diet on brain perfusion in older adults. Nitric. Oxide 24, 34–42. doi: 10.1016/j.niox.2010.10.002 20951824 PMC3018552

[B138] Qin L. Z. Liu X. B. Sun Q. F. Fan Z. P. Xia D. S. Ding G.. (2012). Sialin (slc17a5) functions as a nitrate transporter in the plasma membrane. Proc. Natl. Acad. Sci. U.S.A. 109, 13434–13439.doi.org/10.1073/pnas.1116633109. doi: 10.1073/pnas.1116633109 22778404 PMC3421170

[B139] Qudeimat M. A. Alyahya A. Karched M. Behbehani J. Salako N. O. (2021). Dental plaque microbiota profiles of children with caries-free and caries-active dentition. J. Dent. 104, 103539. doi: 10.1016/j.jdent.2020.103539 33248211

[B140] RajendraA.BondonnoN. P.MurrayK.ZhongL.Rainey-SmithS. R.GardenerS. L.. (2025). Baseline habitual dietary nitrate intake and alzheimer’s disease related neuroimaging biomarkers in the Australian imaging, biomarkers and lifestyle study of ageing. J. Prev. Alzheimers Dis. 11, 100161. doi: 10.1016/j.tjpad.2025.100161 40221237

[B141] Raubenheimer K. Bondonno C. Blekkenhorst L. Wagner K. H. Peake J. M. Neubauer O. (2019). Effects of dietary nitrate on inflammation and immune function, and implications for cardiovascular health. Nutr. Rev. 77, 584–599. doi: 10.1093/nutrit/nuz025 31150091

[B142] Reher V. G. Zenóbio E. G. Costa F. O. Reher P. Soares R. V. (2007). Nitric oxide levels in saliva increase with severity of chronic periodontitis. J. Oral. Sci. 49, 271–276. doi: 10.2334/josnusd.49.271 18195509

[B143] Reichardt E. Eigenthaler M. Jost-Brinkmann P. G. Stellzig-Eisenhauer A. Verna C. Plumeier I.. (2025). Influence of orthodontic appliances and nitrate on the oral microbiota. Appl. Microbiol. Biotechnol. 109, 111. doi: 10.1007/s00253-025-13496-0 40328933 PMC12055954

[B144] Reinders C. A. Jonkers D. Janson E. A. Stockbrügger R. W. Stobberingh E. E. Hellström P. M.. (2007). Rectal nitric oxide and fecal calprotectin in inflammatory bowel disease. Scand. J. Gastroenterol. 42, 1151–1157. doi: 10.1080/00365520701320505 17852876

[B145] Ren T. Roy R. Knowles R. (2000). Production and consumption of nitric oxide by three methanotrophic bacteria. Appl. Environ. Microbiol. 66, 3891–3897. doi: 10.1128/aem.66.9.3891-3897.2000 10966405 PMC92235

[B146] Rocha B. S. Correia M. G. Pereira A. Henriques I. Da Silva G. J. Laranjinha J. (2019). Inorganic nitrate prevents the loss of tight junction proteins and modulates inflammatory events induced by broad-spectrum antibiotics: A role for intestinal microbiota? Nitric. Oxide 88, 27–34. doi: 10.1016/j.niox.2019.04.001 30980891

[B147] Rocha B. S. Laranjinha J. (2020). Nitrate from diet might fuel gut microbiota metabolism: Minding the gap between redox signaling and inter-kingdom communication. Free Radic. Biol. Med. 149, 37–43. doi: 10.1016/j.freeradbiomed.2020.02.001 32045656

[B148] Rocha B. S. Nunes C. Laranjinha J. (2016). Tuning constitutive and pathological inflammation in the gut via the interaction of dietary nitrate and polyphenols with host microbiome. Int. J. Biochem. Cell Biol. 81, 393–402. doi: 10.1016/j.biocel.2016.10.021 27989963

[B149] Rojas-Tapias D. F. Brown E. M. Temple E. R. Onyekaba M. A. Mohamed A. M. T. Duncan K.. (2022). Inflammation-associated nitrate facilitates ectopic colonization of oral bacterium veillonella parvula in the intestine. Nat. Microbiol. 7, 1673–1685. doi: 10.1038/s41564-022-01224-7 36138166 PMC9728153

[B150] Rosier B. T. Buetas E. Moya-Gonzalvez E. M. Artacho A. Mira A. (2020a). Nitrate as a potential prebiotic for the oral microbiome. Sci. Rep. 10, 12895. doi: 10.1038/s41598-020-69931-x 32732931 PMC7393384

[B151] Rosier B. T. Moya-Gonzalvez E. M. Corell-Escuin P. Mira A. (2020b). Isolation and characterization of nitrate-reducing bacteria as potential probiotics for oral and systemic health. Front. Microbiol. 11, 555465. doi: 10.3389/fmicb.2020.555465 33042063 PMC7522554

[B152] Rosier B. T. Palazón C. García-Esteban S. Artacho A. Galiana A. Mira A. (2021). A single dose of nitrate increases resilience against acidification derived from sugar fermentation by the oral microbiome. Front. Cell Infect. Microbiol. 11. doi: 10.3389/fcimb.2021.692883 PMC8238012 34195102

[B153] Rosier B. T. Takahashi N. Zaura E. Krom B. P. MartÍnez-Espinosa R. M. van Breda S. G. J.. (2022). The importance of nitrate reduction for oral health. J. Dent. Res. 101, 887–897. doi: 10.1177/00220345221080982 35196931

[B154] Rowland S. N. James L. J. O’Donnell E. Bailey S. J. (2024). Influence of acute dietary nitrate supplementation timing on nitrate metabolism, central and peripheral blood pressure and exercise tolerance in young men. Eur. J. Appl. Physiol. 124, 1381–1396. doi: 10.1007/s00421-023-05369-z 38040982 PMC11055761

[B155] Saijo F. Milsom A. B. Bryan N. S. Bauer S. M. Vowinkel T. Ivanovic M.. (2010). On the dynamics of nitrite, nitrate and other biomarkers of nitric oxide production in inflammatory bowel disease. Nitric. Oxide 22, 155–167. doi: 10.1016/j.niox.2009.11.009 20005300

[B156] Sanchez G. A. Miozza V. A. Delgado A. Busch L. (2014). Total salivary nitrates and nitrites in oral health and periodontal disease. Nitric. Oxide-Biology Chem. 36, 31–35. doi: 10.1016/j.niox.2013.10.012 24211765

[B157] Schirmer M. Denson L. Vlamakis H. Franzosa E. A. Thomas S. Gotman N. M.. (2018). Compositional and temporal changes in the gut microbiome of pediatric ulcerative colitis patients are linked to disease course. Cell Host Microbe 24, 600–610.e604. doi: 10.1016/j.chom.2018.09.009 30308161 PMC6277984

[B158] Schreiber F. Stief P. Gieseke A. Heisterkamp I. M. Verstraete W. de Beer D.. (2010). Denitrification in human dental plaque. BMC Biol. 8, 24. doi: 10.1186/1741-7007-8-24 20307293 PMC2859859

[B159] Seenivasagan R. Rajakumar S. Kasimani R. Ayyasamy P. M. (2014). Screening of assimilatory and dissimilatory denitrifying microbes isolated from nitrate-contaminated water and soil. Prep Biochem. Biotechnol. 44, 586–597. doi: 10.1080/10826068.2013.835734 24499363

[B160] Shakil M. H. Trisha A. T. Rahman M. Talukdar S. Kobun R. Huda N.. (2022). Nitrites in cured meats, health risk issues, alternatives to nitrites: A review. Foods 11, 3355. doi: 10.3390/foods11213355 36359973 PMC9654915

[B161] Shepherd A. I. Gilchrist M. Winyard P. G. Jones A. M. Hallmann E. Kazimierczak R.. (2015). Effects of dietary nitrate supplementation on the oxygen cost of exercise and walking performance in individuals with type 2 diabetes: A randomized, double-blind, placebo-controlled crossover trial. Free Radic. Biol. Med. 86, 200–208. doi: 10.1016/j.freeradbiomed.2015.05.014 25998421

[B162] Siervo M. Lara J. Ogbonmwan I. Mathers J. C. (2013). Inorganic nitrate and beetroot juice supplementation reduces blood pressure in adults: A systematic review and meta-analysis. J. Nutr. 143, 818–826. doi: 10.3945/jn.112.170233 23596162

[B163] Sindelar J. J. Milkowski A. L. (2012). Human safety controversies surrounding nitrate and nitrite in the diet. Nitric. Oxide 26, 259–266. doi: 10.1016/j.niox.2012.03.011 22487433

[B164] Singh M. Teles F. Uzel N. G. Papas A. (2021). Characterizing microbiota from sjögren’s syndrome patients. JDR Clin. Trans. Res. 6, 324–332. doi: 10.1177/2380084420940623 32689841 PMC8209841

[B165] Sobko T. Reinders C. I. Jansson E. A. Norin E. Midtvedt T. Lundberg J. O. (2005). Gastrointestinal bacteria generate nitric oxide from nitrate and nitrite. Nitric. Oxide-Biology Chem. 13, 272–278. doi: 10.1016/j.niox.2005.08.002 16183308

[B166] Sobko T. Reinders C. Norin E. Midtvedt T. Gustafsson L. E. Lundberg J. O. (2004). Gastrointestinal nitric oxide generation in germ-free and conventional rats. Am. J. Physiology-Gastrointestinal Liver Physiol. 287, G993–G997. doi: 10.1152/ajpgi.00203.2004 15256364

[B167] Sowton A. P. Holzner L. M. W. Krause F. N. Baxter R. Mocciaro G. Krzyzanska D. K.. (2025). Chronic inorganic nitrate supplementation does not improve metabolic health and worsens disease progression in mice with diet-induced obesity. Am. J. Physiol. Endocrinol. Metab. 328, E69–e91. doi: 10.1152/ajpendo.00256.2024 39653040 PMC7617849

[B168] Sparacino-Watkins C. Stolz J. F. Basu P. (2014). Nitrate and periplasmic nitrate reductases. Chem. Soc. Rev. 43, 676–706. doi: 10.1039/c3cs60249d 24141308 PMC4080430

[B169] Srihirun S. Park J. W. Teng R. Sawaengdee W. Piknova B. Schechter A. N. (2020). Nitrate uptake and metabolism in human skeletal muscle cell cultures. Nitric. Oxide 94, 1–8. doi: 10.1016/j.niox.2019.10.005 31604144 PMC7341890

[B170] Stewart V. (1994). Regulation of nitrate and nitrite reductase synthesis in enterobacteria. Antonie Van Leeuwenhoek 66, 37–45. doi: 10.1007/bf00871631 7747939

[B171] Stewart O. A. Wu F. Chen Y. (2020). The role of gastric microbiota in gastric cancer. Gut Microbes 11, 1220–1230. doi: 10.1080/19490976.2020.1762520 32449430 PMC7524314

[B172] Stojanovic M. Scepanovic L. Hrncic D. Rasic-Markovic A. Djuric D. Stanojlovic O. (2015). Multidisciplinary approach to nitric oxide signaling: Focus on the gastrointestinal and the central nervous system. Vojnosanit Pregl 72, 619–624. doi: 10.2298/VSP131025051S 26364456

[B173] Sundqvist M. L. Lundberg J. O. Weitzberg E. (2016). Effects of antiseptic mouthwash on resting metabolic rate: A randomized, double-blind, crossover study. Nitric. Oxide-Biology Chem. 61, 38–44. doi: 10.1016/j.niox.2016.10.003 27769826

[B174] Sun-Edelstein C. Mauskop A. (2009). Foods and supplements in the management of migraine headaches. Clin. J. Pain 25, 446–452. doi: 10.1097/AJP.0b013e31819a6f65 19454881

[B175] Tanner A. C. Mathney J. M. Kent R. L. Chalmers N. I. Hughes C. V. Loo C. Y.. (2011). Cultivable anaerobic microbiota of severe early childhood caries. J. Clin. Microbiol. 49, 1464–1474. doi: 10.1128/jcm.02427-10 21289150 PMC3122858

[B176] Timby N. Domellöf M. Hernell O. Lönnerdal B. Nihlen C. Johanssson I.. (2020). Effects of age, sex and diet on salivary nitrate and nitrite in infants. Nitric. Oxide 94, 73–78. doi: 10.1016/j.niox.2019.10.012 31682925

[B177] Tiso M. Schechter A. N. (2015). Nitrate reduction to nitrite, nitric oxide and ammonia by gut bacteria under physiological conditions. PloS One, 10, e0119712. doi: 10.1371/journal.pone.0127490 PMC4372352 25803049

[B178] Topcu Ali O. Akalin F. A. Sahbazoglu K. B. Yamalik N. Kilinc K. Karabulut E.. (2014). Nitrite and nitrate levels of gingival crevicular fluid and saliva in subjects with gingivitis and chronic periodontitis. J. Oral. Maxillofac. Res. 5, e5. doi: 10.5037/jomr.2014.5205 PMC4115597 25089177

[B179] Tribble G. D. Angelov N. Weltman R. Wang B. Y. Eswaran S. V. Gay I. C.. (2019). Frequency of tongue cleaning impacts the human tongue microbiome composition and enterosalivary circulation of nitrate. Front. Cell. Infection Microbiol. 9. doi: 10.3389/fcimb.2019.00039 PMC6406172 30881924

[B180] Tripodi G. Lombardo M. Kerav S. Aiello G. Baldelli S. (2025). Nitric oxide in parkinson’s disease: The potential role of dietary nitrate in enhancing cognitive and motor health via the nitrate-nitrite-nitric oxide pathway. Nutrients 17, 393. doi: 10.3390/nu17030393 39940251 PMC11819985

[B181] Tseng Y. C. Yang H. Y. Lin W. T. Chang C. B. Chien H. C. Wang H. P.. (2021). Salivary dysbiosis in sjögren’s syndrome and a commensal-mediated immunomodulatory effect of salivary gland epithelial cells. NPJ Biofilms Microbiomes 7, 21. doi: 10.1038/s41522-021-00192-w 33707430 PMC7952914

[B182] Vanhatalo A. Bailey S. J. Blackwell J. R. DiMenna F. J. Pavey T. G. Wilkerson D. P.. (2010). Acute and chronic effects of dietary nitrate supplementation on blood pressure and the physiological responses to moderate-intensity and incremental exercise. Am. J. Physiol. Regul. Integr. Comp. Physiol. 299, R1121–R1131. doi: 10.1152/ajpregu.00206.2010 20702806

[B183] Vanhatalo A. Blackwell J. R. L’Heureux J. E. Williams D. W. Smith A. van der Giezen M.. (2018). Nitrate-responsive oral microbiome modulates nitric oxide homeostasis and blood pressure in humans. Free Radic. Biol. Med. 124, 21–30. doi: 10.1016/j.freeradbiomed.2018.05.078 29807159 PMC6191927

[B184] Vanhatalo A. L’Heureux J. E. Kelly J. Blackwell J. R. Wylie L. J. Fulford J.. (2021). Network analysis of nitrate-sensitive oral microbiome reveals interactions with cognitive function and cardiovascular health across dietary interventions. Redox Biol. 41, 101933. doi: 10.1016/j.redox.2021.101933 33721836 PMC7970425

[B185] van Loon A. J. Botterweck A. A. Goldbohm R. A. Brants H. A. van den Brandt P. A. (1997). Nitrate intake and gastric cancer risk: Results from the Netherlands cohort study. Cancer Lett. 114, 259–261. doi: 10.1016/s0304-3835(97)04677-6 9103306

[B186] van Loon A. J. Botterweck A. A. Goldbohm R. A. Brants H. A. van Klaveren J. D. van den Brandt P. A. (1998). Intake of nitrate and nitrite and the risk of gastric cancer: A prospective cohort study. Br. J. Cancer 78, 129–135. doi: 10.1038/bjc.1998.454 9662263 PMC2062934

[B187] vanMaanen J. M. vanGeel A. A. Kleinjans J. C. (1996). Modulation of nitrate-nitrite conversion in the oral cavity. Cancer Detect Prev. 20, 590–596. 8939344

[B188] Velmurugan S. Gan J. M. Rathod K. S. Khambata R. S. Ghosh S. M. Hartley A.. (2016). Dietary nitrate improves vascular function in patients with hypercholesterolemia: A randomized, double-blind, placebo-controlled study. Am. J. Clin. Nutr. 103, 25–38. doi: 10.3945/ajcn.115.116244 26607938 PMC4691670

[B189] Venturelli M. Pedrinolla A. Boscolo Galazzo I. Fonte C. Smania N. Tamburin S.. (2018). Impact of nitric oxide bioavailability on the progressive cerebral and peripheral circulatory impairments during aging and alzheimer’s disease. Front. Physiol. 9. doi: 10.3389/fphys.2018.00169 PMC5861210 29593548

[B190] Vermeiren J. Van de Wiele T. Verstraete W. Boeckx P. Boon N. (2009). Nitric oxide production by the human intestinal microbiota by dissimilatory nitrate reduction to ammonium. J. Biomedicine Biotechnol. 2009, 284718. doi: 10.1155/2009/284718 PMC2771278 19888436

[B191] Wang W. L. Hu L. Chang S. M. Ma L. S. Li X. C. Yang Z.. (2020). Total body irradiation-induced colon damage is prevented by nitrate-mediated suppression of oxidative stress and homeostasis of the gut microbiome. Nitric. Oxide-Biology Chem. 102, 1–11. doi: 10.1016/j.niox.2020.05.002 32470598

[B192] Wang X. Liu H. Yue M. Wang J. Zhang C. Qin L.. (2024). Dietary nitrate maintains intestinal epithelia homeostasis in aged mice. Biogerontology 25, 1171–1187. doi: 10.1007/s10522-024-10127-5 39162978 PMC11486781

[B193] Wang J. Qi J. Zhao H. He S. Zhang Y. Wei S.. (2013). Metagenomic sequencing reveals microbiota and its functional potential associated with periodontal disease. Sci. Rep. 3, 1843. 1843 doi: 10.1038/srep01843 23673380 PMC3654486

[B194] Webb A. J. Patel N. Loukogeorgakis S. Okorie M. Aboud Z. Misra S.. (2008). Acute blood pressure lowering, vasoprotective, and antiplatelet properties of dietary nitrate via bioconversion to nitrite. Hypertension 51, 784–790. doi: 10.1161/hypertensionaha.107.103523 18250365 PMC2839282

[B195] Wei Y. S. Hsiao Y. C. Su G. W. Chang Y. R. Lin H. P. Wang Y. S.. (2020). Identification of hyperglycemia-associated microbiota alterations in saliva and gingival sulcus. Arch. Biochem. Biophys. 682, 108278. doi: 10.1016/j.abb.2020.108278 31981541

[B196] Weiner C. P. Lizasoain I. Baylis S. A. Knowles R. G. Charles I. G. Moncada S. (1994). Induction of calcium-dependent nitric oxide synthases by sex hormones. Proc. Natl. Acad. Sci. U.S.A. 91, 5212–5216. doi: 10.1073/pnas.91.11.5212 7515189 PMC43962

[B197] Weitzberg E. Lundberg J. O. (2013). Novel aspects of dietary nitrate and human health. Annu. Rev. Nutr. 33, 129–159. doi: 10.1146/annurev-nutr-071812-161159 23642194

[B198] Wicaksono D. P. Washio J. Abiko Y. Domon H. Takahashi N. (2020). Nitrite production from nitrate and its link with lactate metabolism in oral veillonella spp. Appl. Environ. Microbiol. 86, e01255-20. doi: 10.1128/aem.01255-20 32769185 PMC7531945

[B199] Wightman E. L. Haskell-Ramsay C. F. Thompson K. G. Blackwell J. R. Winyard P. G. Forster J.. (2015). Dietary nitrate modulates cerebral blood flow parameters and cognitive performance in humans: A double-blind, placebo-controlled, crossover investigation. Physiol. Behav. 149, 149–158. doi: 10.1016/j.physbeh.2015.05.035 26037632

[B200] Willmott T. Ormesher L. McBain A. J. Humphreys G. J. Myers J. E. Singh G.. (2023). Altered oral nitrate reduction and bacterial profiles in hypertensive women predict blood pressure lowering following acute dietary nitrate supplementation. Hypertension 80, 2397–2406. doi: 10.1161/hypertensionaha.123.21263 37702047

[B201] Woessner M. Smoliga J. M. Tarzia B. Stabler T. Van Bruggen M. Allen J. D. (2016). A stepwise reduction in plasma and salivary nitrite with increasing strengths of mouthwash following a dietary nitrate load. Nitric. Oxide 54, 1–7. doi: 10.1016/j.niox.2016.01.002 26778277

[B202] Xia D. Deng D. Wang S. (2003a). Alterations of nitrate and nitrite content in saliva, serum, and urine in patients with salivary dysfunction. J. Oral. Pathol. Med. 32, 95–99. doi: 10.1034/j.1600-0714.2003.00109.x 12542832

[B203] Xia D. S. Deng D. J. Wang S. L. (2003b). Destruction of parotid glands affects nitrate and nitrite metabolism. J. Dent. Res. 82, 101–105. doi: 10.1177/154405910308200205 12562881

[B204] Xu Y. Jia Y. H. Chen L. Huang W. M. Yang D. Q. (2018). Metagenomic analysis of oral microbiome in young children aged 6–8 years living in a rural isolated chinese province. Oral. Dis. 24, 1115–1125. doi: 10.1111/odi.12871 29667264

[B205] Xu Y. Sa Y. Zhang C. Wang J. Shao Q. Liu J.. (2024). A preventative role of nitrate for hypoxia-induced intestinal injury. Free Radic. Biol. Med. 213, 457–469. doi: 10.1016/j.freeradbiomed.2024.01.030 38281627

[B206] Yang X. He L. Yan S. Chen X. Que G. (2021). The impact of caries status on supragingival plaque and salivary microbiome in children with mixed dentition: A cross-sectional survey. BMC Oral. Health 21, 319. doi: 10.1186/s12903-021-01683-0 34172026 PMC8229229

[B207] Yassaghi Y. Jeddi S. Yousefzadeh N. Kashfi K. Ghasemi A. (2023). Long-term inorganic nitrate administration protects against myocardial ischemia-reperfusion injury in female rats. BMC Cardiovasc. Disord. 23, 411. doi: 10.1186/s12872-023-03425-2 37605135 PMC10441752

[B208] Yoo W. Zieba J. K. Foegeding N. J. Torres T. P. Shelton C. D. Shealy N. G.. (2021). High-fat diet-induced colonocyte dysfunction escalates microbiota-derived trimethylamine n-oxide. Science 373, 813–818. doi: 10.1126/science.aba3683 34385401 PMC8506909

[B209] Zhang S. Clasen F. Cai H. Do T. Shoaie S. Carpenter G. H. (2025). Nitrate supplementation affects taste by changing the oral metabolome and microbiome. NPJ Biofilms Microbiomes 11, 69. doi: 10.1038/s41522-025-00689-8 40316518 PMC12048645

[B210] Zhang G. Han H. Zhuge Z. Dong F. Jiang S. Wang W.. (2021a). Renovascular effects of inorganic nitrate following ischemia-reperfusion of the kidney. Redox Biol. 39, 101836. doi: 10.1016/j.redox.2020.101836 33360353 PMC7772560

[B211] Zhang Y. Jia S. B. Li F. Li S. S. Zhang L. J. Tan K. X.. (2021b). Salivary biochemical indices related to early childhood caries. Hua Xi Kou Qiang Yi Xue Za Zhi 39, 300–305. doi: 10.7518/hxkq.2021.03.009 34041879 PMC8218263

